# Safety of GLP-1 Receptor Agonists and Other Second-Line Antidiabetics in Early Pregnancy

**DOI:** 10.1001/jamainternmed.2023.6663

**Published:** 2023-12-11

**Authors:** Carolyn E. Cesta, Ran Rotem, Brian T. Bateman, Gabriel Chodick, Jacqueline M. Cohen, Kari Furu, Mika Gissler, Krista F. Huybrechts, Lars J. Kjerpeseth, Maarit K. Leinonen, Laura Pazzagli, Helga Zoega, Ellen W. Seely, Elisabetta Patorno, Sonia Hernández-Díaz

**Affiliations:** 1 Centre for Pharmacoepidemiology, Department of Medicine Solna, Karolinska Institute, Stockholm, Sweden; 2 Department of Epidemiology, Harvard T.H. Chan School of Public Health, Boston, Massachusetts; 3 Maccabitech Institute for Research and Innovation, Maccabi Healthcare Services; 4 Department of Anesthesiology, Perioperative and Pain Medicine, Stanford University School of Medicine, Stanford, California; 5 Department of Chronic Diseases, Norwegian Institute of Public Health, Oslo, Norway; 6 Centre for Fertility and Health, Norwegian Institute of Public Health, Oslo, Norway; 7 Department of Knowledge Brokers Finnish Institute for Health and Welfare, Helsinki, Finland; 8 Region Stockholm, Academic Primary Health Care Centre, Stockholm, Sweden; 9 Karolinska Institutet, Department of Molecular Medicine and Surgery, Stockholm, Sweden; 10 Research Centre for Child Psychiatry, University of Turku, Turku, Finland; 11 Division of Pharmacoepidemiology and Pharmacoeconomics, Department of Medicine, Brigham and Women’s Hospital and Harvard Medical School, Boston, Massachusetts; 12 Clinical Epidemiology Division, Department of Medicine Solna, Karolinska Institute, Stockholm, Sweden; 13 School of Population Health, Faculty of Medicine & Health, UNSW Sydney, Sydney, New South Wales, Australia; 14 Centre of Public Health Sciences, Faculty of Medicine, University of Iceland, Reykjavik, Iceland; 15 Endocrinology, Diabetes and Hypertension, Brigham and Women’s Hospital and Harvard Medical School, Boston, Massachusetts

## Abstract

**Importance:**

Increasing use of second-line noninsulin antidiabetic medication (ADM) in pregnant individuals with type 2 diabetes (T2D) may result in fetal exposure, but their teratogenic risk is unknown.

**Objective:**

To evaluate periconceptional use of second-line noninsulin ADMs and whether it is associated with increased risk of major congenital malformations (MCMs) in the infant.

**Design, Setting, and Participants:**

This observational population-based cohort study used data from 4 Nordic countries (2009-2020), the US MarketScan Database (2012-2021), and the Israeli Maccabi Health Services database (2009-2020). Pregnant women with T2D were identified and their live-born infants were followed until up to 1 year after birth.

**Exposure:**

Periconceptional exposure was defined as 1 or more prescription fill of sulfonylureas, dipeptidyl peptidase 4 (DPP-4) inhibitors, glucagon-like peptide 1 (GLP-1) receptor agonists, and sodium-glucose cotransporter 2 (SGLT2) inhibitors, or insulin (active comparator) from 90 days before pregnancy to end of first trimester.

**Main Outcomes and Measures:**

Relative risks (RRs) and 95% CIs for MCMs were estimated using log-binomial regression models, adjusting for key confounders in each cohort and meta-analyzed.

**Results:**

Periconceptional exposure to second-line noninsulin ADMs differed between countries (32, 295, and 73 per 100 000 pregnancies in the Nordics, US, and Israel, respectively), and increased over the study period, especially in the US. The standardized prevalence of MCMs was 3.7% in all infants (n = 3 514 865), 5.3% in the infants born to women with T2D (n = 51 826), and among infants exposed to sulfonylureas was 9.7% (n = 1362); DPP-4 inhibitors, 6.1% (n = 687); GLP-1 receptor agonists, 8.3% (n = 938); SGLT2 inhibitors, 7.0% (n = 335); and insulin, 7.8% (n = 5078). Compared with insulin, adjusted RRs for MCMs were 1.18 (95% CI, 0.94-1.48), 0.83 (95% CI, 0.64-1.06), 0.95 (95% CI, 0.72-1.26), and 0.98 (95% CI, 0.65-1.46) for infants exposed to sulfonylureas, DPP-4 inhibitors, GLP-1 receptor agonists, and SGLT2 inhibitors, respectively.

**Conclusions and Relevance:**

Use of second-line noninsulin ADMs is rapidly increasing for treatment of T2D and other indications, resulting in an increasing number of exposed pregnancies. Although some estimates were imprecise, results did not indicate a large increased risk of MCMs above the risk conferred by maternal T2D requiring second-line treatment. Although reassuring, confirmation from other studies is needed, and continuous monitoring will provide more precise estimates as data accumulate.

## Key Points

**Question:**

Is periconceptional use of glucagon-like peptide 1 (GLP-1) receptor agonists or other noninsulin second-line antidiabetic medications (ADMs) associated with increased risk of major congenital malformations?

**Findings:**

This multinational population-based cohort study of more than 50 000 pregnant women with type 2 diabetes and their infants did not find greater risk of malformations after periconceptional use of sulfonylureas, dipeptidyl peptidase 4 inhibitors, GLP-1 receptor agonists, or sodium-glucose cotransporter 2 inhibitors compared with insulin.

**Meaning:**

Use of GLP-1 receptor agonists and other noninsulin second-line ADMs has increased in pregnancy and in this first large study on their teratogenic risk in humans, results provide initial reassurance of their safety.

## Teaser

This cohort study examines the association of periconceptional use of glucagon-like peptide 1 (GLP-1) receptor agonists or other noninsulin second-line antidiabetic medications with increased risk of major congenital malformations.

## Introduction

Type 2 diabetes (T2D) is an increasingly common condition in female individuals of reproductive age,^1,2^ which has resulted in increased use of antidiabetic medication (ADM) during pregnancy.^3,4^ In the general nonpregnant population, metformin is often the first-line pharmacological treatment for T2D, and insulin or other second-line noninsulin ADMs, including sulfonylureas, dipeptidyl peptidase 4 (DPP-4) inhibitors, glucagon-like peptide 1 (GLP-1) receptor agonists, and sodium-glucose cotransporter 2 (SGLT2) inhibitors, can be switched to or added to maintain glycemic control if needed.^5^ Notably, the use of second-line noninsulin ADM has increased in the last decade.^6,7^

For patients with T2D who are planning pregnancy or who are already pregnant, the guideline-recommended treatment has traditionally been insulin, due to the limited data on the safety of noninsulin ADM for fetal development.^8^ However, the intentional use of metformin during pregnancy has become more common and unintended exposure to second-line noninsulin ADM medications during the first trimester, although still rare, has also increased over time.^3,4^ Unintentional pregnancy exposure arises because a proportion of pregnancies are unplanned,^9,10^ and therefore the discontinuation of these medications often occurs during or after organogenesis. Hence, studies are urgently needed to be able to advise patients, clinicians, and regulatory bodies on the potential teratogenic risk of these medications.

To generate evidence on the teratogenic risk of second-line noninsulin ADM, we combined data from 6 large population-based health care databases from 4 Nordic countries, the US, and Israel to identify a cohort of pregnant women with pharmacologically treated T2D around the time of conception. First, we described the time trends of second-line noninsulin ADM use in pregnancy over time. Next, we compared the risk of major congenital malformations (MCMs) overall, and cardiac MCMs specifically, in infants born to women with periconceptional use of second-line noninsulin ADM vs insulin.

## Methods

### Data Sources and Pregnancy Cohorts

This study was conducted within the International Pregnancy Safety Study (InPreSS) Consortium, a collaboration among research groups in several countries, including the Nordic countries, the US, and Israel, all of whom have access to high-quality prospectively collected health care databases and registers.^11^

The Nordic cohort was derived from nationwide population registers and included all pregnancies resulting in singleton live-born infants in Finland, Iceland, Norway, and Sweden from 2009 to the end of available data in each country (2016-2020). The individual-level data from the 4 countries were pooled and harmonized using a common data model.^12^ Information on the Nordic population health registers is available in eMethods 1 in Supplement 1.

The US cohort consisted of commercially insured pregnant women linked to their live-born (singleton and multiple) infants included in the MarketScan Research Database (2012-2021), one of the largest national health care administration databases.^13^ To ensure capture of all diagnosis codes and prescription fills during the study period, pregnant women were required to have continuous insurance coverage from at least 6 months before pregnancy to 1 month after delivery; infants were required to have coverage from birth until 90 days after birth, unless they died sooner.

The Israeli cohort consisted of pregnancies resulting in a singleton live-born infant (2010-2020) from women continuously enrolled for at least 1 year preconception in the Maccabi Health Services (MHS) database; infants were required to have at least 1 year of complete follow-up postbirth, unless they died sooner. MHS is Israel’s second-largest health care organization serving as both insurer and health care service to approximately 25% of the Israeli population.

Pregnancies with a diagnosis of a fetal chromosomal abnormality or with exposure to a known teratogenic medication (eTable 1 in Supplement 1) were excluded from each cohort (eFigure in Supplement 1).

### Ethical Approval

Use of the Nordic data was approved by applicable ethics review boards and/or data providing authorities (eMethods 1 in Supplement 1). Use of the MarketScan data was approved by the institutional review board at the Harvard T.H. Chan School of Public Health. Use of the Israeli MHS data was approved by the institutional review boards at MHS and Harvard T.H. Chan School of Public Health, which granted a waiver of informed consent.

### Study Population

The study population included pregnancies in women with pregestational T2D linked to live-born infants. In the US data, T2D was identified using a validated algorithm based on diagnoses and medication prescription fills. In the validation study, the algorithm had a positive predictive value of 87% compared with electronic medical records^14^; however, this estimate was likely conservative when applied to the present study where the exposures of interest, second-line noninsulin ADMs were indicated almost exclusively for T2D during the study period. The algorithm was adapted for use in the Nordic and MHS data, and optimized based on the best available information, including laboratory measurements in MHS. Algorithms and criteria are described in detail in eMethods 2 in Supplement 1. In this study, we refer to biologic sex and use the term *pregnant woman* to define pregnant human females of any gender identity. Gender identity was not recorded in the databases.

### Exposure

Periconceptional exposure was defined based on the filling of 1 or more prescriptions of the respective drug class (eTable 2 in Supplement 1) from 90 days before the first day of the last menstrual period (LMP) to the end of the first trimester because drug supplies for chronic illness often cover 1 to 3 months. A secondary exposure definition for sensitivity analyses required the filling of 1 or more prescriptions from LMP to the end of the first trimester.

Pregnancies were then classified into the following exposure groups: periconceptional use of no ADM, metformin only, insulin (with or without coprescriptions of metformin, but no other ADM), sulfonylureas, DPP-4 inhibitors, GLP-1 receptor agonists, or SGLT2 inhibitors. Women in the latter 4 exposure groups were allowed to have coprescriptions of any other ADM during the periconception period.

### Major Congenital Malformations

The presence of any MCM overall and the subgroup of major cardiac malformations were identified using diagnosis and procedure codes as described in detail for each cohort in eTables 3 and 4 in Supplement 1. Briefly, MCMs were defined using infant diagnoses from the date of birth to 1 year after birth in the Nordic and Israeli cohorts, and using claims in the infant and the women’s records from date of birth to 90 days after birth in the US cohort.^15^

### Covariates

Key baseline characteristics were described for pregnant women with periconceptional use of the second-line ADM, including maternal age, comorbidities (ie, obesity, hypertension, cardiovascular disease, diabetic complications, polycystic ovary syndrome [PCOS]) and other prescription medication (ie, antihypertensive medication, lipid modifying agents; defined in eTable 5 in Supplement 1). Hemoglobin A_1c_ (HbA_1c_) levels, which are a measure of glycemic control over the previous 3 months, were available for a subset of pregnancies in the US and Israeli cohorts (eTable 6 in Supplement 1). The mean (SD) and median (IQR) were calculated for each exposure group (HbA_1c_ unit = %) with linked laboratory test results for HbA_1c_ levels from between 90 days before LMP to the end of the first trimester.

### Statistical Analysis

Analyses were conducted separately in the pooled Nordic cohort, the US cohort, and the Israeli cohort. We calculated the proportion of pregnancies with periconceptional exposure to second-line noninsulin ADM by birth year. The standardized weighted prevalence of any MCM and cardiac malformations were calculated for all exposure groups to account for regional differences in both utilization of ADM classes and baseline frequency of MCMs. Within each cohort, the crude and adjusted relative risks (RRs) with 95% CIs were estimated for each of the 4 second-line noninsulin exposure groups compared with insulin using a log-binomial model. In the adjusted model, maternal age, year of birth, obesity, and specific Nordic country (in the pooled Nordic cohort only) were added. The crude and adjusted estimates from the Nordic, US, and Israeli cohorts were then combined using fixed effect meta-analysis using R statistical software (version 4.3.1, R Foundation). The analysis was completed on June 23, 2023.

## Results

### Study Population and Exposure Groups

**Figure 1.**
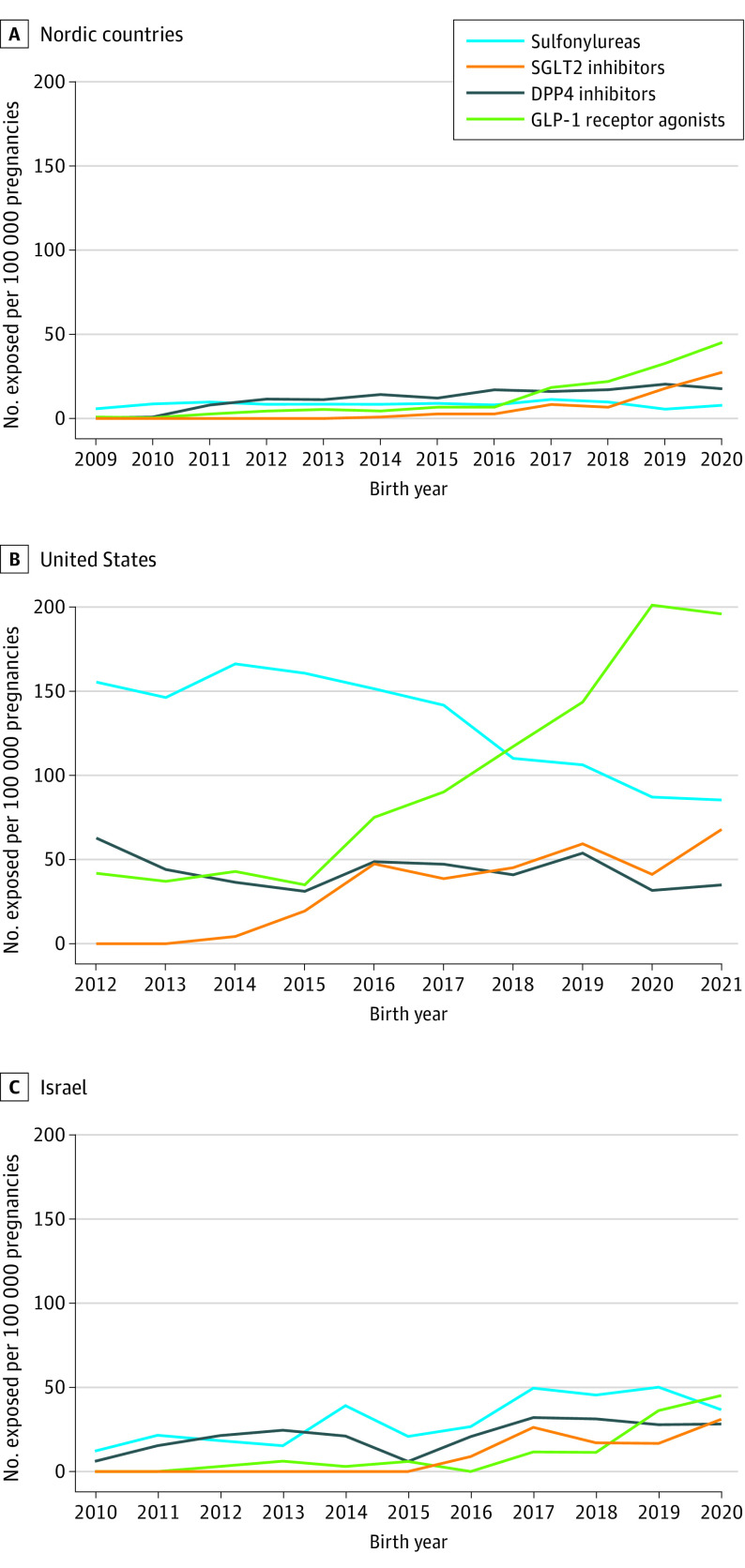
Prevalence of Periconceptional Second-Line Noninsulin Antidiabetic Medication Exposure Over Time in the Nordics, US, and Israel The denominator contains all live-born infants per year per database. Nordic results are based on pooled data from national health registers in Finland, Iceland, Norway, and Sweden. US results based on data from the MarketScan database. Israel results based on data from Maccabi Health Service database. DPP-4 indicates dipeptidyl peptidase 4; GLP-1, glucagon-like peptide-1; SGLT2, sodium-glucose cotransporter 2.

In a total of 3 514 865 pregnancies from the 3 data sources combined, 51 826 (1.5%) were in women with pregestational T2D, of whom 15 148 (29.2%) were treated with ADM in the periconceptional period (Nordics, 9693; US, 4778; Israel, 677) (eFigure in Supplement 1). Among these pregnancies, 7440 (50%) used metformin only, 5078 (34%) insulin, 1352 (9.0%) sulfonylureas, 687 (4.5%) DPP-4 inhibitors, 938 (6.2%) GLP-1 receptor agonists, and 335 (2.2%) SGLT2 inhibitors. Periconceptional use of second-line noninsulin ADM increased over time, particularly in the US for GLP-1 receptor agonists, and except for sulfonylureas, which remained low in the Nordic countries, decreased in the US, and increased slightly in Israel (Figure 1). Use of other ADM classes such as glitazones, meglitinides, and α-glucosidase inhibitors remained very low.

### Cohort Characteristics

Table 1 shows the baseline maternal characteristics, by study cohort, for women with pregestational T2D in the 4 second-line noninsulin ADM and insulin exposure groups. Compared with those using insulin, women using second-line noninsulin ADMs were slightly younger in the US and Israel and slightly older in the Nordic countries. The prevalence of obesity and PCOS was highest in women using GLP-1 receptor agonists; chronic hypertension, cardiovascular disease, and use of antihypertensive and lipid-modifying agents were highest in SGLT2 inhibitor users. Diabetic complications were highest among women using insulin and SGLT2 inhibitors. Among second-line noninsulin ADM users, coprescription fills of insulin were common (37%-82%), as were coprescription fills of metformin (25%-90%).

**Table 1.** Selected Characteristics of Pregnant Women With Pregestational Type 2 Diabetes and Periconceptional Use of Second-Line Antidiabetic Medication^a^

Characteristic	Nordic countries					US					Israel				
	Insulin	Sulfonylureas	DPP-4 inhibitors	GLP-1 receptor agonists	SGLT2 inhibitors	Insulin	Sulfonylureas	DPP-4 inhibitors	GLP-1 receptor agonists	SGLT2 inhibitors	Insulin	Sulfonylureas	DPP-4 inhibitors	GLP-1 receptor agonists	SGLT2 inhibitors
Total pregnancies, No.	3269	198	266	214	82	1387	1049	341	681	218	422	115	80	43	35
Maternal age at delivery, y, %															
<30	28.8	27.3	18.0	31.3	22.0	12.5	16.9	16.4	17.8	13.3	8.8	15.7	15.0	20.9	11.4
30-34	32.6	26.3	33.8	34.6	28.1	36.2	35.7	34.0	33.9	34.9	30.3	26.1	18.8	27.9	25.7
35-39	27.3	29.8	33.5	22.4	34.2	36.2	33.5	36.4	36.3	36.7	32.5	32.2	25.0	27.9	31.4
≥40	11.4	16.7	14.7	11.7	15.9	15.1	14.0	13.2	12.0	15.1	28.4	26.1	41.3	23.3	31.4
Comorbidities and comedication, %															
Overweight/ obesity	43.9	42.4	54.9	60.3	54.9	33.4	28.3	32.6	47.9	45.4	39.1	25.2	33.8	65.1	45.7
Chronic hypertension	10.9	14.1	15.4	13.6	13.4	29.1	28.6	35.8	28.9	38.5	18.0	11.3	25.0	11.6	11.4
Diabetic complications	14.2	17.7	16.5	22.4	28.1	12.3	5.7	9.7	7.6	18.8	28.0	13.0	20.0	11.6	22.9
Cardiovascular disease	0.5	0.0	NA^b^	NA^b^	17.1	0.8	0.4	2.3	0.6	1.4	4.3	1.7	3.8	0	2.9
Polycystic ovary syndrome	9.3	8.1	11.3	22.0	16.9	10.9	7.3	10.0	19.4	12.4	24.2	18.3	23.8	20.9	17.1
Antihypertensive drugs	7.1	10.6	12.8	12.2	9.8	17.2	20.4	27.9	25.3	38.5	15.9	8.7	12.5	18.6	25.7
Lipid modifying agents	4.0	12.6	13.2	11.2	17.1	5.6	9.2	15.5	12.2	21.1	9.2	8.7	28.8	20.9	40.0
Other ADM prescription fills, %															
Insulin	100	74.2	67.7	62.2	81.7	100	36.6	58.4	38.5	61.0	100	33.9	67.5	44.2	68.6
Metformin	35.9	65.2	63.2	55.6	59.8	61.7	56.8	81.2	49.3	81.2	46	25.2	90.0	39.5	71.4
Sulfonylureas	0	100	12.0	4.2	7.3	0	100	24.9	7.6	20.6	0	100	8.8	9.3	2.9
DPP-4 inhibitors	0	9.6	100	2.3	19.5	0	8.1	100	4.3	16.5	0	6.1	100	4.7	25.7
GLP-1 receptor agonists	0	4.6	1.9	100	11.0	0	5.0	8.5	100	22.9	0	3.5	2.5	100	11.4
SGLT2 inhibitors	0	2.5	6.0	4.2	100	0	4.3	10.6	7.3	100	0	0.9	11.3	NA^b^	100

Abbreviations: ADM, antidiabetic medication; DPP-4, dipeptidyl peptidase 4; GLP-1, glucagon-like peptide-1; SGLT2, sodium-glucose cotransporter 2.

^a^ Separately for the Nordic countries (pooled Finland, Iceland, Norway, Sweden); US, MarketScan; and Israel, Maccabi Health Services databases.

^b^ Percentages based on counts <5 are not shown for data privacy policies in the Nordic countries.

For the subsample of pregnant women in the US (n = 397) and Israel (n = 575) with available laboratory data, the median periconceptional HbA_1c_ levels were highest among those using either insulin or the second-line noninsulin ADM, particularly DPP-4 inhibitors and SGLT2 inhibitors, relative to other pregnant women with T2D treated with metformin or not treated pharmacologically (eTable 6 in Supplement 1).

### Prevalence of Malformations

**Figure 2.**
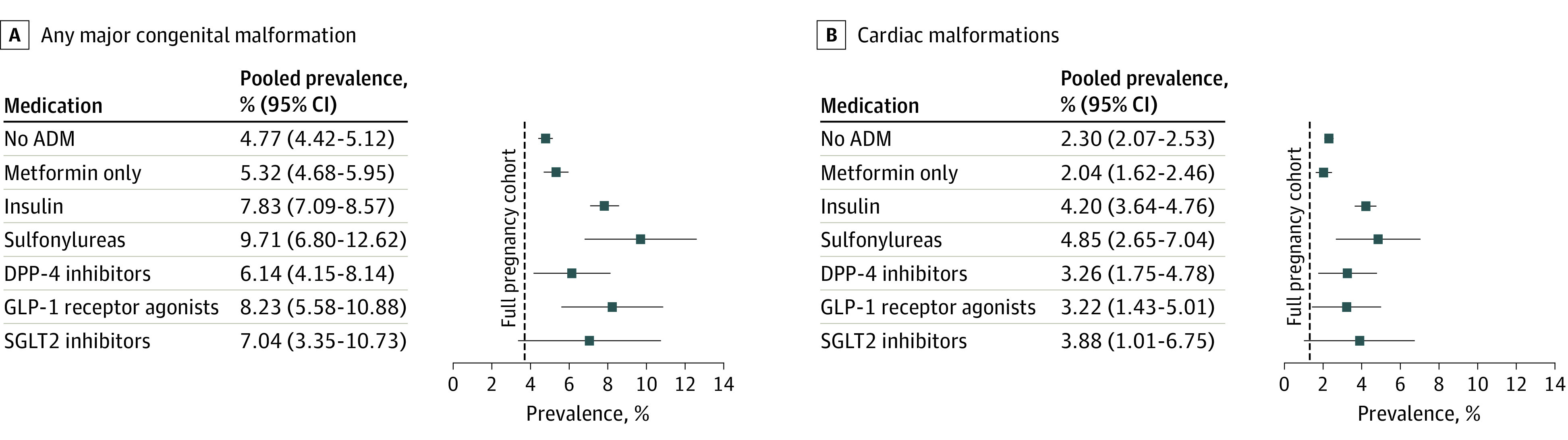
Prevalence of Any and Cardiac Major Congenital Malformations in Infants With Exposure to Maternal Periconception Type 2 Diabetes and Antidiabetic Medication Use The corresponding number of exposed, number of cases, prevalence, and 95% CIs combined and for each study cohort are reported in eTable 7 in Supplement 1. ADM indicates antidiabetic medication; DPP-4, dipeptidyl peptidase 4; GLP-1, glucagon-like peptide-1; SGLT2, sodium-glucose cotransporter 2.

Figure 2 (and eTable 7 in Supplement 1) shows the prevalence of any MCM and cardiac malformations for each exposure group. As a reference, there were 132 283 infants born with an MCM in the full pregnancy cohort (3.76%), and 2584 infants born with an MCM in the study population of women with T2D (5.28%). Within the study population, the prevalence of MCMs was lower among infants with periconceptional exposure to no ADM (4.77%) or metformin only (5.32%) than among those exposed to insulin (7.83%), sulfonylureas (9.71%), DPP-4 inhibitors (6.14%), GLP-1 receptor agonists (8.23%), or SGLT2 inhibitors (7.04%). For cardiac malformations, the prevalence was similarly elevated among infants born to women with T2D (2.25% vs 1.31% in the full pregnancy cohort) and lower among infants with periconceptional exposure to no ADM (2.30%) or metformin only (2.04%) than among those exposed to insulin (4.20%), sulfonylureas (4.85%), DPP-4 inhibitors (3.26%), GLP-1 receptor agonists (3.22%), and SGLT2 inhibitors (3.88%).

### Relative Risks of Malformations

Table 2 presents the meta-analyzed crude and adjusted RRs (aRRs). Compared with infants with periconceptional exposure to insulin, the aRRs were compatible with no substantial increased risk for MCMs among infants with periconceptional exposure to sulfonylureas (aRR, 1.18; 95% CI, 0.94-1.48), DPP-4 inhibitors (aRR, 0.83; 95% CI, 0.64-1.06), GLP-1 receptor agonists (aRR, 0.95; 95% CI, 0.72-1.26), or SGLT2 inhibitors (aRR, 0.98; 95% CI, 0.65-1.46). Similarly, results did not suggest an increased risk for cardiac malformations after periconceptional exposure to any of the second-line noninsulin ADMs studied compared with insulin. Individual cohort RRs are presented in eTable 8 in Supplement 1.

**Table 2.** Risk for Any and Cardiac Major Congenital Malformations in Infants Born to Women With Type 2 Diabetes and Periconceptional Use of Second-Line Noninsulin Antidiabetic Medications Compared With Insulin^a^

Treatment	No. of exposed cases/No. of exposed (%)^b^	Crude relative risk (95% CI)	Adjusted relative risk (95% CI)^c^
**Any major congenital malformation**			
Insulin	400/5078 (7.8)	1 [Reference]	1 [Reference]
Sulfonylureas	121/1362 (9.7)	1.14 (0.91-1.42)	1.18 (0.94-1.48)
DPP-4 inhibitors	50/687 (6.1)	0.91 (0.67-1.24)	0.83 (0.64-1.06)
GLP-1 receptor agonists	75/938 (8.2)	1.02 (0.78-1.33)	0.95 (0.72-1.26)
SGLT2 inhibitors	30/335 (7.0)	1.13 (0.76-1.67)	0.98 (0.65-1.46)^d^
**Cardiac malformations**			
Insulin	212/5078 (4.2)	1 [Reference]	1 [Reference]
Sulfonylureas	50/1362 (4.8)	1.05 (0.75-1.47)	1.05 (0.75-1.48)
DPP-4 inhibitors	24/687 (3.3)	0.91 (0.59-1.41)	0.90 (0.58-1.39)
GLP-1 receptor agonists	23/938 (3.2)	0.67 (0.42-1.06)	0.68 (0.42-1.12)
SGLT2 inhibitors	15/335 (3.9)	1.22 (0.70-2.13)	1.10 (0.63-1.92)^d^

Abbreviations: DPP-4, dipeptidyl peptidase 4; GLP-1, glucagon-like peptide-1; SGLT2, sodium-glucose cotransporter 2.

^a^ Individual study cohort estimates are reported in eTable 8 in Supplement 1.

^b^ Standardized prevalence.

^c^ Adjusted for birth year, maternal age, obesity, and specific Nordic country (in the pooled Nordic cohort only; Finland, Iceland, Norway, Sweden).

^d^ US model only adjusted for birth year and obesity.

Restricting the exposure definition in the sensitivity analysis to the filling of 1 or more prescriptions in the first trimester resulted in fewer exposed infants: 1070 to sulfonylureas, 400 to DPP-4 inhibitors, 461 to GLP-1 receptor agonists, and 181 to SGLT2 inhibitors. Consistent with the main analysis, the crude RR estimates did not suggest an increased risk for any MCM after exposure to any of the second-line noninsulin ADMs compared with insulin: sulfonylureas (RR, 1.02; 95% CI, 0.79-1.32), DPP-4 inhibitors (RR, 0.82; 95% CI, 0.55-1.23), GLP-1 receptor agonists (RR, 1.03; 95% CI, 0.73-1.47), or SGLT2 inhibitors (RR, 1.20; 95% CI, 0.69-2.11). Results were also similar to the main analysis for cardiac malformations (eTable 9 in Supplement 1).

## Discussion

In this cohort study by the InPreSS consortium including more than 50 000 pregnancies in women with pregestational T2D from 6 countries, we observed no elevated risk of MCMs after periconceptional exposure to GLP-1 receptor agonists or any of the second-line noninsulin ADM classes evaluated compared with insulin, another second-line ADM and the traditional treatment for T2D in pregnancy. Further, we showed an increase in periconceptional use of second-line noninsulin ADMs, particularly GLP-1 receptor agonists in the US, highlighting that there has been a shift in how T2D in reproductive-aged women is treated. Although this study did not suggest that these medications have strong teratogenic effects, there is a need for further research to fully evaluate the safety of these medications in pregnancy.

T2D is an increasingly common condition in female individuals of reproductive age, and consequently in pregnant patients.^1,2^ In-line with previous studies, we found an elevated prevalence (5.3%) of MCMs in infants born to women with pregestational T2D, compared with the general population (3.7%).^16,17,18^ The effect of T2D is believed to be at least partially mediated by hyperglycemia because poor glycemic control during pregnancy is associated with an increased risk of MCMs and other adverse pregnancy outcomes.^19,20,21,22^ This supports the importance of glycemic control and having safe and effective medications available during pregnancy.^23^

Although insulin does not cross the placenta and is considered nonteratogenic,^23^ little to no data are available on what risks, if any, noninsulin ADMs may pose when used during the time of embryogenesis. However, use of metformin may be considered according to some guidelines.^24^ Because metformin use in pregnant women with T2D has increased over time^3,4^ and is also used for treatment of infertility and PCOS, there is some information on the safety of metformin exposure during the first trimester.^25,26^ We observed that in all 3 study cohorts, use of DPP-4 inhibitors, GLP-1 receptor agonists, and SGLT2 inhibitors has also increased rapidly over the last decade, mimicking their use in the general population of patients with T2D.^6,7^ Notably, use of GLP-1 receptor agonists has increased substantially, particularly in the US, among individuals with T2D, likely due to their weight loss effects. With the recent approval for specific medications within this class to be used as anti-obesity treatment,^27^ the number of infants prenatally exposed to this class of medications will presumably continue to increase.

### Strengths and Limitations

To reduce confounding by indication, we restricted our analysis to women with T2D who had used second-line ADMs periconceptionally and compared noninsulin vs insulin treatments, much like a hypothetical clinical trial would have pregestational T2D as an inclusion criterion and use of insulin as an active comparator. We have previously shown^28^ the importance of comparing ADM strategies used for treating similar severity of T2D in pregnancy to achieve balance in markers of glycemic control between comparison groups (eg, HbA_1c_) and reduce confounding by the underlying diabetes progression.

Although the study design optimized clinical equipoise between comparison groups, residual bias due to the channeling of T2D patients with specific characteristics to specific second-line ADM treatments is expected because these medications are recommended based on the presence of comorbidities such as obesity, cardiac, and kidney diseases. The clinical characteristics (eg, comorbidities and comedication use) of the exposure groups were in-line with these treatment recommendations. Confounding by obesity and cardiovascular conditions would preferentially affect GLP-1 receptor agonists and SGLT2 inhibitors, and bias the RR estimates for MCMs upward. Reassuringly, adjusting for obesity in this study did not substantially affect the RRs, and given the expected direction of confounding, adjustment for additional and maternal comorbidities would likely attenuate the estimates toward the null. Moreover, the HbA_1c_ levels were slightly higher in some of the second-line noninsulin ADM groups compared with insulin, indicating that confounding by glycemic control could, if anything, bias the RR for MCMs upward. Despite the likely overestimation of the RRs due to residual confounding, they were most compatible with a null effect relative to insulin.

Filled prescriptions around conception might not result in exposure during embryogenesis, particularly for those filled before LMP. Among second-line noninsulin ADM users, coprescription fills of insulin or metformin were common in the periconceptional period, indicating scenarios that are difficult to disentangle in our data: concomitant use or adherence to guideline recommendations for switching to metformin or insulin. If there are increased risks conferred by use of second-line noninsulin ADM throughout the first trimester, then early pregnancy switching among the exposed groups could lead to an underestimation of those risks. We conducted a sensitivity analysis including only pregnant women with prescription fills during the first trimester. Fewer pregnancies were included in this analysis, yet the conclusion that there was not a substantial increased risk of MCMs overall or cardiac malformations remained.

The study population was restricted to pregnancies resulting in live births because information on MCM was not available or was only partially available for pregnancies that resulted in stillbirth, miscarriage, or termination. Conditioning on livebirth might introduce selection bias and potentially underestimate the RRs only if MCMs were more lethal or preferentially terminated in pregnancies exposed to specific ADMs relative to those exposed to insulin; however, to our knowledge, there are currently no studies that have investigated whether there is evidence toward this. Evaluating the potential effect of noninsulin ADM on fertility, miscarriages, or pregnancy termination is challenging and beyond the scope of this study.

Despite including data from 6 countries, the number of infants exposed to specific second-line noninsulin ADM classes remained low during the study period and the estimates, thus, imprecise with the upper limits of the 95% CI including up to a 2-fold increased risk. Although this study is valuable because there is no available information on the teratogenicity of these medications in humans, and results are reassuring that these drugs are not major teratogens, confirmation from other studies is needed. Because the use of these medications is becoming more common for treatment of T2D and for other indications (ie, obesity), the number of infants with prenatal exposure will increase.

## Conclusions

In this study, infants born to women with pregestational T2D were associated with having a higher prevalence of MCMs, including cardiac malformations, compared with infants in the general population. However, in infants born to women with T2D treated with second-line ADM, we did not observe a greater risk of MCMs after periconceptional exposure to sulfonylureas, DPP-4 inhibitors, GLP-1 receptor agonists, or SGLT2 inhibitors compared with insulin. Although reassuring, confirmation from other studies is needed, and continuous monitoring will provide more precise risk estimates in the future as data accumulate.

## Notes

Supplement 1. eFigure 1. Flow chart outlining the derivation of the study population eTable 1. Known Teratogenic drugs eTable 2. Antidiabetic Medications eTable 3. Definition of Major Congenital Malformations of Interest in Nordic Countries eTable 4. Definition of Major Congenital Malformations of Interest in MarketScan Research Database and Maccabi Health Services Database eTable 5. Definition and Assessment Period of Covariates of Interest eTable 6. Mean and Median HbA1c for the full study population and women with pre-gestational type 2 diabetes mellitus with different periconceptional antidiabetic medication use in a subset of the US MarketScan and Israel Maccabi Health Services databases eTable 7. Prevalence and 95% confidence intervals of any and cardiac major congenital malformation in live-born infants in the full pregnancy cohort and born to women with type 2 diabetes mellitus with periconceptional use of no antidiabetic medication, metformin only, insulin, or 2nd-line non-insulin antidiabetic medications eTable 8. Crude and adjusted relative risks and 95% confidence intervals for any and cardiac major congenital malformations in infants born to women with type 2 diabetes mellitus and periconceptional use of 2nd-line non-insulin antidiabetic medication compared with insulin, presented separately for the Nordic countries (pooled Finland, Iceland, Norway, Sweden), US MarketScan, and Israel Maccabi Health Services databases eTable 9. Crude relative risks and 95% confidence intervals for any and cardiac major congenital malformation in infants born to women with type 2 diabetes mellitus and first trimester use of 2nd-line non-insulin antidiabetic medications compared with insulin, presented meta-analyzed and separately for the Nordic countries (pooled Finland, Iceland, Norway, Sweden), US MarketScan, and Israel Maccabi Health Services databases eMethods 1. Description of Nordic Data and Ethical Approval Information eMethods 2. Pre-gestational Diabetes Algorithms eReferences

Supplement 2. Data Sharing Statement

## References

[1] Deputy NP, Kim SY, Conrey EJ, Bullard KM. Prevalence and changes in preexisting diabetes and gestational diabetes among women who had a live birth - United States, 2012-2016. MMWR Morb Mortal Wkly Rep. 2018;67(43):1201-1207. doi:10.15585/mmwr.mm6743a2 30383743 PMC6319799

[2] Cho NH, Shaw JE, Karuranga S, . IDF Diabetes Atlas: Global estimates of diabetes prevalence for 2017 and projections for 2045. Diabetes Res Clin Pract. 2018;138:271-281. doi:10.1016/j.diabres.2018.02.023 29496507

[3] Cesta CE, Cohen JM, Pazzagli L, . Antidiabetic medication use during pregnancy: an international utilization study. BMJ Open Diabetes Res Care. 2019;7(1):e000759. doi:10.1136/bmjdrc-2019-000759 31798900 PMC6861111

[4] Wood ME, Patorno E, Huybrechts KF, . The use of glucose-lowering medications for the treatment of type 2 diabetes mellitus during pregnancy in the United States. Endocrinol Diabetes Metab. 2022;5(2):e00319. doi:10.1002/edm2.319 34953068 PMC8917861

[5] American Diabetes Association. 9. Pharmacologic approaches to glycemic treatment: *Standards of Medical Care in Diabetes-2021.* Diabetes Care. 2021;44(suppl 1):S111-S124. doi:10.2337/dc21-S009 33298420

[6] Pottegård A, Andersen JH, Søndergaard J, Thomsen RW, Vilsbøll T. Changes in the use of glucose-lowering drugs: a Danish nationwide study. Diabetes Obes Metab. 2023;25(4):1002-1010. doi:10.1111/dom.14947 36514856

[7] Shin H, Schneeweiss S, Glynn RJ, Patorno E. Trends in first-line glucose-lowering drug use in adults with type 2 diabetes in light of emerging evidence for SGLT-2i and GLP-1RA. Diabetes Care. 2021;44(8):1774-1782. doi:10.2337/dc20-2926 34385345 PMC8385465

[8] American Diabetes Association. 14. Management of diabetes in pregnancy: *Standards of Medical Care in Diabetes-2021.* Diabetes Care. 2021;44(suppl 1):S200-S210. doi:10.2337/dc21-S014 33298425

[9] Finer LB, Henshaw SK. Disparities in rates of unintended pregnancy in the United States, 1994 and 2001. Perspect Sex Reprod Health. 2006;38(2):90-96. doi:10.1363/3809006 16772190

[10] Bearak J, Popinchalk A, Ganatra B, . Unintended pregnancy and abortion by income, region, and the legal status of abortion: estimates from a comprehensive model for 1990-2019. Lancet Glob Health. 2020;8(9):e1152-e1161. doi:10.1016/S2214-109X(20)30315-6 32710833

[11] Huybrechts KF, Straub L, Karlsson P, . Association of in utero antipsychotic medication exposure with risk of congenital malformations in Nordic countries and the US. JAMA Psychiatry. 2023;80(2):156-166. doi:10.1001/jamapsychiatry.2022.4109 36477338 PMC9856848

[12] Cohen JM, Cesta CE, Kjerpeseth L, . A common data model for harmonization in the Nordic Pregnancy Drug Safety Studies (NorPreSS). Nor Epidemiol. 2021;29(1-2):117-123. doi:10.5324/nje.v29i1-2.4053

[13] MacDonald SC, Cohen JM, Panchaud A, McElrath TF, Huybrechts KF, Hernández-Díaz S. Identifying pregnancies in insurance claims data: methods and application to retinoid teratogenic surveillance. Pharmacoepidemiol Drug Saf. 2019;28(9):1211-1221. doi:10.1002/pds.4794 31328328 PMC6830505

[14] Wood ME, Chen ST, Huybrechts KF, . Validation of a claims-based algorithm to identify pregestational diabetes among pregnant women in the United States. Epidemiology. 2021;32(6):855-859. doi:10.1097/EDE.0000000000001397 34183529 PMC8478806

[15] Palmsten K, Huybrechts KF, Kowal MK, Mogun H, Hernández-Díaz S. Validity of maternal and infant outcomes within nationwide Medicaid data. Pharmacoepidemiol Drug Saf. 2014;23(6):646-655. doi:10.1002/pds.3627 24740606 PMC4205050

[16] Dolk H, Loane M, Garne E. The prevalence of congenital anomalies in Europe. Adv Exp Med Biol. 2010;686:349-364. doi:10.1007/978-90-481-9485-8_20 20824455

[17] Centers for Disease Control and Prevention (CDC). Update on overall prevalence of major birth defects–Atlanta, Georgia, 1978-2005. MMWR Morb Mortal Wkly Rep. 2008;57(1):1-5. 18185492

[18] Ornoy A, Reece EA, Pavlinkova G, Kappen C, Miller RK. Effect of maternal diabetes on the embryo, fetus, and children: congenital anomalies, genetic and epigenetic changes and developmental outcomes. Birth Defects Res C Embryo Today. 2015;105(1):53-72. doi:10.1002/bdrc.21090 25783684

[19] Arendt LH, Pedersen LH, Pedersen L, . Glycemic control in pregnancies complicated by pre-existing diabetes mellitus and congenital malformations: a Danish population-based study. Clin Epidemiol. 2021;13:615-626. doi:10.2147/CLEP.S298748 34345185 PMC8325058

[20] Davidson AJF, Park AL, Berger H, . Association of improved periconception hemoglobin A1c with pregnancy outcomes in women with diabetes. JAMA Netw Open. 2020;3(12):e2030207. doi:10.1001/jamanetworkopen.2020.30207 33355674 PMC7758806

[21] Cea-Soriano L, García-Rodríguez LA, Brodovicz KG, Masso Gonzalez E, Bartels DB, Hernández-Díaz S. Safety of non-insulin glucose-lowering drugs in pregnant women with pre-gestational diabetes: a cohort study. Diabetes Obes Metab. 2018;20(7):1642-1651. doi:10.1111/dom.13275 29498473

[22] Davidson AJF, Park AL, Berger H, . Risk of severe maternal morbidity or death in relation to elevated hemoglobin A1c preconception, and in early pregnancy: a population-based cohort study. PLoS Med. 2020;17(5):e1003104. doi:10.1371/journal.pmed.1003104 32427997 PMC7236974

[23] Kitzmiller JL, Ferrara A, Peng T, Cissell MA, Kim C. Preexisting Diabetes and Pregnancy. In: Cowie CC, Casagrande SS, Menke A, , eds. Diabetes in America. Bethesda (MD) no conflicts of interest. National Institute of Diabetes and Digestive and Kidney Diseases; 2018. 33651557

[24] National Institute for Health and Care Excellence. Guidelines In: Diabetes in pregnancy: management from preconception to the postnatal period. London: National Institute for Health and Care Excellence (NICE) Copyright NICE 2020. 2020. 32212588

[25] Abolhassani N, Winterfeld U, Kaplan YC, . Major malformations risk following early pregnancy exposure to metformin: a systematic review and meta-analysis. BMJ Open Diabetes Res Care. 2023;11(1):e002919. doi:10.1136/bmjdrc-2022-002919 36720508 PMC9890805

[26] Kjerpeseth LJ, Cesta CE, Furu K, . Metformin versus insulin and risk of major congenital malformations in pregnancies with type 2 diabetes: a Nordic register-based cohort study. Diabetes Care. 2023;46(8):1556-1564. doi:10.2337/dc23-0256 37343541

[27] Bessesen DH, Van Gaal LF. Progress and challenges in anti-obesity pharmacotherapy. Lancet Diabetes Endocrinol. 2018;6(3):237-248. doi:10.1016/S2213-8587(17)30236-X 28919062

[28] Cesta CE, Hernández-Díaz S, Huybrechts KF, . Achieving comparability in glycemic control between antidiabetic treatment strategies in pregnancy when using real world data. Pharmacoepidemiol Drug Saf. 2023. doi:10.1002/pds.5665 37461243 PMC10792121

