# Supplementary material for: Safety of GLP-1 Receptor Agonists and Other Second-Line Antidiabetics in Early Pregnancy
Source: JAMA Intern Med. 2023 Dec 11;184(2):144–52. doi: 10.1001/jamainternmed.2023.6663 (PMC10714281; doi:10.1001/jamainternmed.2023.6663)
Supplement: Supplement 1. — eFigure 1. Flow chart outlining the derivation of the study population eTable 1. Known Teratogenic drugs eTable 2. Antidiabetic Medications eTable 3. Definition of Major Congenital Malformations of Interest in Nordic Countries eTable 4. Definition of Major Congenital Malformations of Interest in MarketScan Research Database and Maccabi Health Services Database eTable 5. Definition and Assessment Period of Covariates of Interest eTable 6. Mean and Median HbA1c for the full study population and women with pre-gestational type 2 diabetes mellitus with different periconceptional antidiabetic medication use in a subset of the US MarketScan and Israel Maccabi Health Services databases eTable 7. Prevalence and 95% confidence intervals of any and cardiac major congenital malformation in live-born infants in the full pregnancy cohort and born to women with type 2 diabetes mellitus with periconceptional use of no antidiabetic medication, metformin only, insulin, or 2nd-line non-insulin antidiabetic medications eTable 8. Crude and adjusted relative risks and 95% confidence intervals for any and cardiac major congenital malformations in infants born to women with type 2 diabetes mellitus and periconceptional use of 2nd-line non-insulin antidiabetic medication compared with insulin, presented separately for the Nordic countries (pooled Finland, Iceland, Norway, Sweden), US MarketScan, and Israel Maccabi Health Services databases eTable 9. Crude relative risks and 95% confidence intervals for any and cardiac major congenital malformation in infants born to women with type 2 diabetes mellitus and first trimester use of 2nd-line non-insulin antidiabetic medications compared with insulin, presented meta-analyzed and separately for the Nordic countries (pooled Finland, Iceland, Norway, Sweden), US MarketScan, and Israel Maccabi Health Services databases eMethods 1. Description of Nordic Data and Ethical Approval Information eMethods 2. Pre-gestational Diabetes Algorithms eReferences [file jamainternmed-e236663-s001.pdf]

Supplemental Online Content

Cesta CE, Rotem R, Bateman BT, et al. Safety of GLP-1 receptor agonists and other second-line antidiabetics in early pregnancy. *JAMA Intern Med*. Published online December 11, 2023. doi:10.1001/jamainternmed.2023.6663

eFigure 1. Flow chart outlining the derivation of the study population
eTable 1. Known Teratogenic drugs
eTable 2. Antidiabetic Medications
eTable 3. Definition of Major Congenital Malformations of Interest in Nordic Countries
eTable 4. Definition of Major Congenital Malformations of Interest in MarketScan Research Database and Maccabi Health Services Database
eTable 5. Definition and Assessment Period of Covariates of Interest
eTable 6. Mean and Median HbA1c for the full study population and women with pre-gestational type 2 diabetes mellitus with different periconceptional antidiabetic medication use in a subset of the US MarketScan and Israel Maccabi Health Services databases
eTable 7. Prevalence and 95% confidence intervals of any and cardiac major congenital malformation in live-born infants in the full pregnancy cohort and born to women with type 2 diabetes mellitus with periconceptional use of no antidiabetic medication, metformin only, insulin, or 2nd-line non-insulin antidiabetic medications
eTable 8. Crude and adjusted relative risks and 95% confidence intervals for any and cardiac major congenital malformations in infants born to women with type 2 diabetes mellitus and periconceptional use of 2nd-line non-insulin antidiabetic medication compared with insulin, presented separately for the Nordic countries (pooled Finland, Iceland, Norway, Sweden), US MarketScan, and Israel Maccabi Health Services databases
eTable 9. Crude relative risks and 95% confidence intervals for any and cardiac major congenital malformation in infants born to women with type 2 diabetes mellitus and first trimester use of 2nd-line non-insulin antidiabetic medications compared with insulin, presented meta-analyzed and separately for the Nordic countries (pooled Finland, Iceland, Norway, Sweden), US MarketScan, and Israel Maccabi Health Services databases
eMethods 1. Description of Nordic Data and Ethical Approval Information
eMethods 2. Pregestational Diabetes Algorithms
eReferences

This supplemental material has been provided by the authors to give readers additional information about their work.

© 2023 Cesta CE, et al. *JAMA Intern Med.*

© 2023 Cesta CE, et al. *JAMA Intern Med.*

Supplementary Figure 1: Flow chart outlining the derivation of the study population

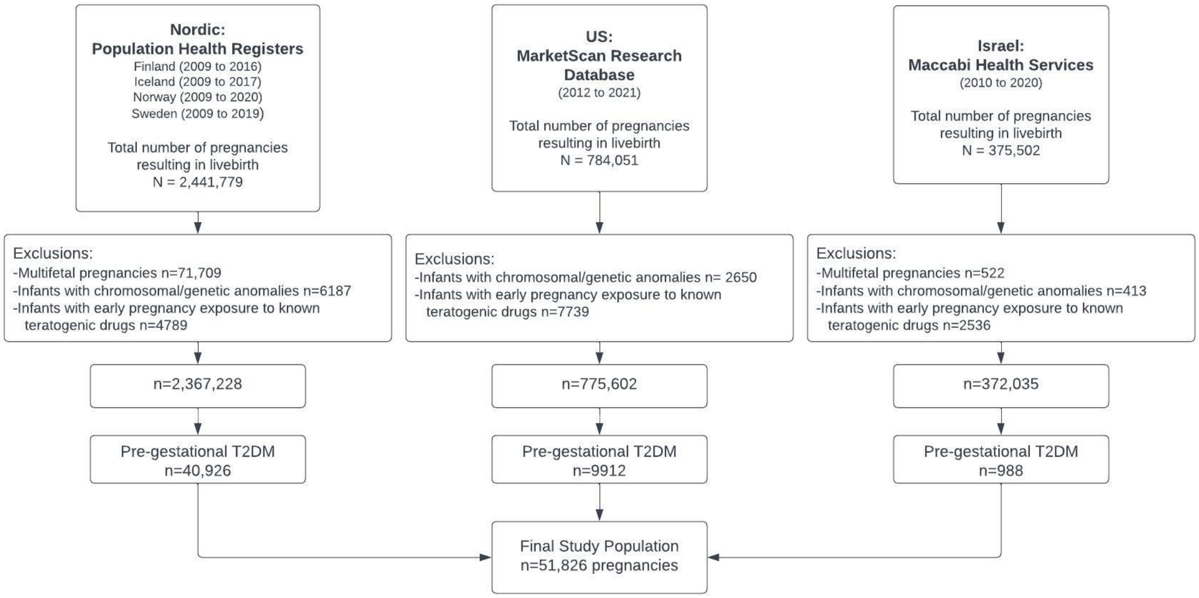

Abbreviations: T2DM, type 2 diabetes mellitus

© 2023 Cesta CE, et al. *JAMA Intern Med.*

Supplementary Table 1. Known Teratogenic drugs

Drug	US MarketScan (identified by generic drug names)	Nordic cohort and Israel-MHS (ATC Codes)
Warfarin	Yes	B01AA03
Antineoplastic drugs:	Yes	L01DA<br>L01AB01<br>L01AA02<br>M04AC01<br>L01AA01<br>L01DB01<br>L01BB02<br>L01BA01, L04AX03<br>L01CA01<br>L01CA02
Lithium	yes	N05AN01
Topical or systemic retinoids, including isotretinoin	yes	D10BA01<br>D10AD54<br>D10BA04<br>D05BB<br>D11AH04
Misoprostol	yes	A02BB01, G02AD06<br>M01AE56<br>M01AB55
Thalidomide	yes	L04AX02
Valproic acid	yes	N03AG01
Carbamazepine	yes	Nordics: not included[a]<br>Israel: N03AF01

[a] based on recent evidence of no teratogenic effect of carbamazepine in Nordic populations(1)

© 2023 Cesta CE, et al. *JAMA Intern Med.*

Supplementary Table 2. Antidiabetic Medications

Antidiabetic Medication Class	US MarketScan Research Database (Generic names)	Nordic cohort and Israel-MHS (ATC Codes)
Insulin	Insulin Aspart Protamine/ Insulin Aspart<br>Insulin Aspart, Recombinant<br>Insulin Aspart/ Insulin Aspart Protamine<br>Insulin Degludec<br>Insulin Degludec/Liraglutide<br>Insulin Detemir<br>Insuline Glargine, Recombinant<br>Insulin Glargine, Recombinant/Lixisenatide<br>Insulin Glulisine<br>Insulin Human Inhaled<br>Insulin Human Isophane<br>Insulin Human Isophane/ Insulan Human Regular<br>Insulin Human Regular<br>Insulin Human Zinc (LEnte)<br>Insulin Lispro Protamine/ Insulin Lispro<br>Insulin Lispro, Recombinant<br>Insulin Lispro/ Insulin Lispro Protamine	A10A
Metformin (only)	Metformin Hydrochloride	A10BA02
Sulfonylureas	Chlorpropamide<br>Glimepiride<br>Glimepiride/Pioglitazone Hydrochloride<br>Glipizide<br>Glipizide/Metformin Hydrochloride<br>Glyburide<br>Glyburide, Micronized<br>Glyburide/Metformin Hydrochloride<br>Tolazamide<br>Tolbutamide<br>Acetohexamide	A10BB<br>A10BD01<br>A10BD02<br>A10BD04<br>A10BD06
Dipeptidyl peptidase 4 inhibitors	Alogliptin Benzoate<br>Alogliptin Benzoate/Metformin Hydrochloride<br>Alogliptin Benzoate/Pioglitazone HCl<br>Empagliflozin/Linagliptin<br>Linagliptin<br>Linagliptin/Metformin Hydrochloride<br>Metformin Hydrochloride/Saxagliptin Hydrochloride<br>Metformin Hydrochloride/Sitagliptin Phosphate<br>Saxagliptin Hydrochloride<br>Simvastatin/Sitagliptin Phosphate<br>Dapagliflozin/Saxagliptin<br>Sitagliptin Phosphate	A10BH<br>A10BD07<br>A10BD08<br>A10BD09<br>A10BD10<br>A10BD11<br>A10BD12<br>A10BD13<br>A10BD18<br>A10BD19<br>A10BD21<br>A10BD24<br>A10BD25<br>A10BD27
Glucagon-like peptide-1 receptor agnists	Albiglutide<br>Dulaglutide<br>Exenatide<br>Liraglutide<br>Insulin Degludec/Liraglutide<br>Lixisenatide<br>Insulin Glargine, Recombinant/Lixisenatide<br>Semaglutide	A10BJ
Sodium-glucose co-transporter 2 inhibitors	Canagliflozin<br>Canagliflozin/Metformin Hydrochloride<br>Dapagliflozin<br>Dapagliflozin/Metformin Hydrochloride<br>Dapagliflozin Propanediol<br>Dapagliflozin Propanediol/Metformin Hydrochloride<br>Dapagliflozin/Saxagliptin Empagliflozin<br>Empagliflozin/Linagliptin<br>Empagliflozin/Metformin Hydrochloride<br>Ertugliflozin	A10BK<br>A10BD15<br>A10BD16<br>A10BD19<br>A10BD20<br>A10BD21<br>A10BD22<br>A10BD23<br>A10BD24<br>A10BD25<br>A10BD27

© 2023 Cesta CE, et al. *JAMA Intern Med.*

Other antidiabetic medication (Alpha glucosidase inhibitors, thiazolidinediones, meglitinides)	Acarbose<br>Miglitol<br>Glimepiride/Pioglitazone Hydrochloride<br>Metformin Hydrochloride/Pioglitazone Hydrochloride<br>Metformin Hydrochloride/Rosiglitazone Maleate<br>Alogliptin Benzoate/Pioglitazone HCl<br>Pioglitazone Hydrochloride<br>Rosiglitazone Maleate<br>Metformin Hydrochloridel/Rosiglitazone Maleat<br>Troglitazone<br>Pramlintide Acetate<br>Nateglinide<br>Repaglinide	A10BF<br>A10BD17<br>A10BG<br>A10BD03<br>A10BD04<br>A10BD05<br>A10BD06<br>A10BD09<br>A10BD12<br>A10BD26<br>A10BX<br>A10BD14

© 2023 Cesta CE, et al. *JAMA Intern Med.*

Supplementary Table 3. Definition of Major Congenital Malformations of Interest in Nordic Countries

Outcomes are defined based on data from the Medical Birth, Malformation and/or Patient Registers from the date of birth to one year after birth.
For diagnoses recorded in the inpatient setting, one code was considered sufficient.
For diagnoses recorded in the outpatient setting, at least 2 codes recorded on different days were required.

Note: While malformation subgroups 1-3 and 5-12 were not considered as separate outcomes in the analyses, we present the definition of these subgroups as they are used to define major congenital malformations overall.

**Subgroup**	**ICD-10**	**ICD-9A (Finland)**[a]
1. Nervous system	Q00-Q07<br>Except (Q04.61, Q07.80, Q07.82)	740-742<br>Except 74280
2. Eye	Q10-Q15<br>Except Q10.1-Q10.3, Q10.5, Q13.5	743<br>Except 74345, 74361-74363, 74365
3. Ear, face, and neck	Q16-Q18<br>Except Q17.0-Q17.5, Q17.9, Q18.0-Q18.2, Q18.4-Q18.7, (Q18.80), Q18.9	744<br>Except 7441, 74420-74424, 74430, 7444, 74480-74483, 7449
4. Cardiac	Q20-Q26<br>Except (Q21.11), Q246, Q250 and preterm, (Q25.41), Q25.6 and preterm, Q26.1	745, 746, 7470-7474<br>Except 74550, 74687, 7470 and preterm, 74723, 747325 and preterm, 74741, 74749
5. Respiratory	Q30, Q32-Q34<br>Except Q30.1-Q30.9, Q32.0, Q32.2, (Q33.00), Q33.1, Q33.6	748<br>Except 7481, 7482-7483 (other), 74840, 74851, 74859, 74862
6. Oro-facial clefts	Q35-Q37<br>Except Q35.7, or if occurring with Q00, Q042	749<br>Except 74908, or if occurring with 740, 74226
7. Digestive system	Q38-Q45, Q79.0<br>Except Q38.1, Q38.2, (Q385.0), Q40.0, Q40.1, (Q40.21), Q43.0, (Q43.20, Q43.81, Q43.82), Q44.4, (Q458.3)	750, 751, 7566<br>Except 75000, 75011-75013, 75024, 75050, 75051, 7506, 75101, 7513, 75166
8. Abdominal wall	Q79.2, Q79.3, Q79.5	75670, 75671, 75679
9. Genital	Q50-Q56<br>Except Q50.1 (Q50.10, Q50.11), Q50.2, Q50.5, Q52.3, Q52.5, Q52.7, Q53, Q54.4, (Q55.20, Q55.21)	752<br>Except 75208, 75211, 75243, 75244, 7525, 75261 (urinary), 752621, 75282, 75286
10. Urinary	Q60-Q64, Q79.4<br>Except Q61.0, (Q61.90 genetic) Q62.7, Q63.3	75261, 753, 75672<br>Except 75310, 75334, 753485
11. Limb[b]	Q66-Q74<br>Except Q66.1-Q66.9, Q67.0-Q67.8, Q68.0, (Q68.10, Q68.21) Q68.3, Q68.4, Q68.5, (Q74.00)	754-755<br>Except 7540-7542 (other), 7543, 75440-75443, 75451-75453, 75459, 7546, 7547, 75480-75482, 755525, 755606, 755616, 755645, 755646, 75566, 75581 (genetic)
12. Other	Q27, Q28, Q31, Q75-Q85, Q89<br>Except Q27.0, Q31.4, Q75.1 (genetic), Q75.2, Q75.3, Q75.4 (genetic), Q76.0, (Q76.43, Q76.60, Q76.62), Q76.5, (Q76.71), Q79.0 (digestive), Q79.2 (abdominal wall), Q79.3 (abdominal wall), Q79.4 (urinary), Q79.5 (abdominal wall), Q82.5, Q82.8, Q83.3, Q84.5, Q84.6, (Q89.11), Q89.9	7475-7479, 7482-7483, 7540-7542, 756, 757, 759<br>Except 7475, 74836, 7540, 7541, 75421, 75604 (genetic), 756085, 75610, 7562, 75630, 75632, 75633, 7566 (digestive), 75670 (abdominal wall), 75671 (abdominal wall), 75672 (urinary), 75679 (abdominal wall), 75686, 7572, 7573, 75751, 75758, 75765, 75902, 75904, 75911, 7598 (genetic), 7599
X. Chromosomal anomaly, genetic syndrome, or teratogenic infection	D82.1, P35.0, P35.1, P37.1, (Q61.90), Q75.1, Q75.4, Q87, Q90-Q99<br>Except Q95	27911, 75581, 75604, 758, 7598, 7710, 7711, 77121<br>Except 7584, 75989
**Any MCM**[c]	**Any of the subgroups 1-12, excluding those in subgroup X**	

The definition of major congenital malformations was based on EUROCAT (EUROCAT [2013]. EUROCAT Guide 1.4: Instruction for the registration of congenital anomalies. EUROCAT Central Registry, University of Ulster. Version 01/12/2020). Codes in parentheses under ICD-10 are minor anomalies coded according to British Paediatric Association (BPA) modification of ICD that cannot be identified with standard ICD-10. The Finnish Register of Congenital Anomalies uses ICD-9A (A=Atlanta or Metropolitan Atlanta Congenital Defects Program [MACDP] which is almost identical to BPA version). [a]It is possible to exclude more minor anomalies with ICD-9A than standard ICD. [b]Due to concerns of low validity, we did not include hip dislocation or dysplasia (ICD-10 Q65; ICD-9A 7543, 75566). [c]Due to concern that we may exclude children exposed to potentially teratogenic medications of interest ICD-10 code Q86 (congenital malformation syndromes due to known exogenous causes, not elsewhere classified) and ICD-9A code 75989 were neither excluded nor included in any subgroup.

© 2023 Cesta CE, et al. *JAMA Intern Med.*

Supplementary Table 4. Definition of Major Congenital Malformations of Interest in MarketScan Research Database and Maccabi Health Services Database

The approach described here has previously been validated to identify several of the described malformations of interest in another US health care database with high positive predictive values.(2, 3)

MarketScan Research Database: Consider claims in infant record between date of birth (DOB) and DOB+90 and/or in the maternal record between delivery and delivery+30, using both in- and out-patient data. Refer to the ICD-9 codes in eTable 2 below.

Maccabi Health Services (MHS) Database: Consider claims in infant records between DOB and DOB+1 year.

For cardiovascular malformations overall:
The outcome is considered present if there are
- ≥ 2 dates with a code for any of the cardiac malformations (regardless of the subgroup) OR
- ≥ 1 date with a code for any of the cardiac malformations* and a cardiac procedure code OR (no produre codes for MHS)
- ≥ 1 date with a code for any of the cardiac malformations* and the infant died within the first 90 days (1 year for MHS).

The outcome is considered maternal if there is ≥1 date with a code of interest in the maternal records between LMP and LMP+105 AND there are no codes in the infant record between DOB and DOB+90.

For non-cardiovascular malformations and malformation subgroups:
The outcome is considered present if there are
- ≥ 2 dates with a code for the malformation group/subgroup OR
- ≥ 1 date with a code for the malformation group/subgroup and an outcome-specific procedure code OR
- ≥ 1 date with a code for the malformation group/subgroup and the infant died within the first 90 days (1 year for MHS).

The outcome is considered maternal if there is ≥1 date with a code of interest in the maternal records between LMP and LMP+105 AND there are no codes in the infant record between DOB and DOB+90.

For any major congenital malformation:
The outcome is considered present if the infant contributed either to any of the non-cardiovascular malformations or to cardiovascular malformations overall.

*the following codes should not be considered: 745.4x, 745.5x, 747.0x, 746.4x, 746.6x, 746.99, 747.3x if preterm, 746.02 if preterm, 747.5x, 416.0x if preterm, 747.83 if preterm, 746.08, 746.105

MALFORMATION GROUP	Malformation Subgroup	ICD-9	ICD-10
1. Cardiovascular Anomalies	Conotruncal Defects	745.0x, 745.1x, 745.2x	Q20.0-Q20.3, Q20.5, Q20.8, Q21.3
	Single Ventricle	745.3x	Q20.4
	Ventricular Septal Defect**	745.4x	Q21.0
	ASD	745.5x AND no preterm[2]	Q21.1 AND no preterm[2]
	AV Septal Defect	745.6x	Q21.2
	Right sided defects	746.00, 746.01, 746.09, 746.1x, 746.2x, 746.83, 747.3x AND no preterm[2], 746.02 AND no preterm[2]	Q22, Q22.0, Q22.1 AND no preterm[2], Q22.3-Q22.9, Q24.3, Q25.5 AND no preterm[2], Q25.6 AND no preterm[2], Q25.7x AND no preterm[2]
	Left sided defects	747.1x, 747.2x, 746.3x, 746.5x, 746.7x, 746.81, 746.82	Q23, Q23.0, Q23.2, Q23.4, Q23.8, Q23.9, Q24.2, Q24.4, Q25.1x-Q25.4x
	PDA	747.0x and no preterm[2]	Q25.0 AND no preterm[2]
	PPHN	(416.0x or 747.83) and no preterm	(I27.0, P29.3x) AND no preterm
	Great cardiac veins	747.4 747.41 747.42	Q26.0-Q26.4
	Other cardiac	745.7x,745.8x, 746.8[1], 746.84-746.89	Q20.6, Q21.4, Q21.8, Q24.0, Q24.1, Q24.5, Q24.6, Q24.8, Q25.8
	Cardiac NOS	745[1], 745.9, 746[1], 746.9x (do not count 746.99), 747[1]	Q20[1], Q20.9, Q21[1], Q21.9, Q24[1], Q24.9, Q25[1], Q25.9≥
2. Oral cleft		749.xx	Q35.x-Q37.x
3. Central Nervous System		740.xx-742.xx	Q00.x-Q07.x
4. Eye Anomalies		743.xx (do not count 743.6x, 743.8x)	Q10[1], Q10.4, Q10.7, Q11.x-Q15.x (do not count Q13.5)
5. Ear Anomalies		744.0x, 744.23, 744.3x	Q16.x-Q17.x (do not count Q17.0, Q17.3, Q17.5)
6. Other vascular (non-cardiac)		747.40, 747.49, 747.6x-747.9x (do not count 747.83)	Q26.5-Q26.9, Q27.x-Q28.x (do not count Q27.0, Q27.4)

© 2023 Cesta CE, et al. *JAMA Intern Med.*

**7. Respiratory malformations**		748.xx (do not count 748.1x, 748.3x)	Q30.0, Q30.1, Q31.0, Q32.x (do not count Q32.0), Q33.x (do not count Q33.1), Q34.x
**8. Gastrointestinal**		750.xx-751.xx (do not count 750.0x, 750.1x, 751.0x)	Q38.x-Q45.x (do not count Q38.1, Q38.2, Q38.3, Q43.0)
**9. Genital (male and female)**	Overall	752.xx (do not count 752.42, 752.52) (in addition, do not count 752.5x if preterm), 756.71	Q50.xx-Q52.xx (do not count Q52.3, Q52.5), Q53.0x, Q53.1xx AND no preterm, Q53.2xx AND no preterm, Q53.9 AND no preterm, Q54.xx-Q56.xx (do not count Q55.22), Q64.0, Q79.4
	Hypospadias	752.61	Q54.x (do not count Q54.4)
	Cryptorchidism	752.5x (do not count 752.52) (in addition, do not count 752.5x if preterm)	Q53.xxx AND no preterm
**10. Urinary**		753.xx (do not count 753.7x), 756.71	Q60.xx-Q64.xx (do not count Q64.0, Q64.4), Q79.4
**11. Musculoskeletal**		754.1x, 754.2x, 756.xx (do not count 756.2x, 756.7x)	Q68.0, Q75.x-Q78.x (do not count Q76.5), Q79.0, Q79.1, Q79.6, Q79.8, Q79.9
**12. Limb defects (includes hip)**		755.xx (do not count 755.65, 755.63), 754.4x-754.8x (do not count 754.81, 754.82)	Q65.81, Q65.82, Q66.xx, Q68.1-Q68.8, Q69.x-Q74.x
**13. Abdominal Wall**		756.7x	Q79.2- Q79.5x
**14. Other**		757.0x, 757.1x, 759.xx (do not count 759.5x, 759.81-759.83)	Q80.8, Q80.9, Q86.x, Q89.xx

[1] Only count this code. Do not count any digits after this code.
[2] In order for this malformation to be present, ≥ 2 dates with a diagnostic code for a malformation of which at least one code is documented at >= 6 weeks after DoB are required.

The 13 groups numbered above present the main classes of malformation. An infant with one of these confirmed malformations (specific inclusion criteria see above) would be considered to have a major malformation, acknowledging that the “other” malformations may include a range of defects and the specificity might not be as high as for others. However, these groups should be relatively small.

A separate group are infants with **chromosomal anomalies and excluded from the study population**. Defined as:

- ≥ 2 dates with a code for a malformation within the group “chromosomal abnormalities”
- 1 date with a code for a malformation within the group and infant death

Chromosomal abnormalities	Dx	ICD9: 758xx, 75981, 75982, 75983	ICD-10: Q87.1, Q87.11 Q87.19 Q87.40, Q87.410, Q87.418, Q87.42, Q87.43, Q90.0, Q90.1, Q90.2, Q90.9, Q91.0, Q91.1, Q91.2, Q91.3, Q91.4, Q91.5, Q91.6, Q91.7, Q92.0, Q921, Q92.2, Q92.5, Q92.61, Q92.62, Q92.7, Q92.8, Q92.9, Q93.0, Q93.1, Q93.2, Q93.3, Q93.4, Q93.5, Q93.7, Q93.81, Q93.88, Q93.89, Q93.9, Q95.0, Q95.1, Q95.2, Q95.3, Q95.5, Q95.8, Q95.9, Q96.0, Q96.1, Q96.2, Q96.3, Q96.4, Q968, Q96.9, Q97.0, Q97.1, Q97.2, Q97.3, Q97.8, Q979, Q980, Q98.1, Q98.3, Q98.4, Q98.5, Q98.6, Q98.7, Q98.8, Q98.9, Q99.0, Q99.1, Q99.2, Q99.8, Q99.9

Supplementary Table 5. Definition and Assessment Period of Covariates of Interest

Condition	US (MarketScan Research Database)	Nordic Registers	Israel (Maccabi Health Services Database)
**Demographic characteristics**			
Year of infant birth	yes	As recorded in birth register	As recorded in the electronic medical record
Maternal age at infant birth	yes	As recorded in birth register	As recorded in the electronic medical record
Country of infant birth	n/a	Finland, Iceland, Norway, Sweden	Israel
**Maternal conditions**	≥1 inpatient or outpatient code in maternal record from LMP-180 to LMP+90	As recorded in patient register or birth register from LMP-365 to LMP+97	Assessment period = LMP-180 to LMP+90
Obesity or overweight	**ICD-9:** 278.0x, 649.1x, V85.3x, V85.4x<br>**ICD-10:** E66.01, E66.09, E66.1, E66.2, E66.3, E66.8, E66.9, O99.210, O99.211, O99.212, O99.213, O99.214, O99.215, Z68.30, Z68.31, Z68.32, Z68.33, Z68.34, Z68.35, Z68.36, Z68.37, Z68.38, Z68.39, Z68.41, Z68.42, Z68.43, Z68.44, Z68.45	BMI≥30 as calculated from height and weight collected in early pregnancy.<br>Available from 2012 for Iceland.	ICD-9: 278.0x<br>or<br>BMI>25 in LMP-365 to LMP+90
Chronic hypertension	**ICD-9:** 401.x-405.x, 642.0x-642.2x, 642.7x, 642.9x<br>**ICD-10:** I1.0, I1.10, I1.19, I1.20, I12.9, I13.0, I13.10, I1.311, I13.2, I15.0, I15.1, I15.2, I15.8, I159, I16.0, I161, I16.9, O10.011, O10.012, O10.013, O10.019, O10.02, O10.03, O10.111, O10.112, O10.113, O10.119, O10.12, O10.13, O10.211, O10.212, O10.213, O10.219, O10.22, O10.23, O10.311, O10.312, O10.313, O10.319, O10.32, O10.33, O10.411, O10.412, O10.413, O10.419, O10.42, O10.43, O10.911, O10.912, O10.913, O10.919, O10.92, O10.93, O11.1, O11.2, O11.3, O11.4, O11.5, O11.9, O16.1, O16.2, O16.3, O16.4, O16.5, O16.9	**ICD-10:** O10, O16, I10-15	Meeting one the following: (1) ICD-9 codes 401.x-405.x; (2) At least 4 abnormal blood pressure measurements (systolic >140, diastolic>90), which account for at least 50% of all available measurements; (3) at least 6 dispensing records in a 3-year period for one of the following classes of medications: thiazides, calcium channel and beta blockers, ACE (angiotensin converting enzyme) inhibitors or ARBs (angiotensin-receptor blockers)
Diabetic complications	**ICD-9:** 583.81, 250.4x, 362.0x, 250.5x, 366.41, 365.44, 362.83, 707.1x, 585.3, 585.4, 585.5, 585.6, 582.xx, 583.xx, 585.1, 585.2, 585.9, 586.xx, 587.xx, 642.1x, 250.4x, 250.40, 250.41, 250.42, 250.43, 403.xx, 404.xx, 572.4x, 580.xx, 584.xx, 580.0x, 580.4x, 580.89, 580.9x, 582.4x, 642.1x, 791.2x, 791.3x<br>**ICD-10:** E08.21, E08.40, E08.41, E08.42, E08.43, E09.21, E11.0, E11.1, E11.21, E11.22, E11.29, E11.31, E11.311, E11.319, E11.32, E11.321, E11.3211, E11.3212, E11.3213, E11.3219, E11.329, E11.3291, E11.3292, E11.3293, E11.3299, E11.33, E11.331, E11.3311, E11.3312, E11.3313, E11.3319, E11.339, E11.3391, E11.3392, E11.3393, E11.3399, E11.34, E11.341, E11.3411, E11.3412, E11.3413, E11.3419, E11.349, E11.3491, E11.3492, E11.3493, E11.3499, E11.35, E11.351, E11.3511, E11.3512, E11.3513, E11.3519, E11.352, E11.3521, E11.3522, E11.3523, E11.3529, E11.353, E11.3531, E11.3532, E11.3533, E11.3539, E11.354, E11.3541, E11.3542, E11.3543, E11.3549, E11.355, E11.3551, E11.3552, E11.3553, E11.3559, E11.359, E11.3591, E11.3592, E11.3593, E11.3599, E11.36, E11.37, E11.37X1, E11.37X2, E11.37X3, E11.37X9, E11.39, E11.40, E11.41, E11.42, E11.43, E11.44, E11.49, E11.610, E11.621, E11.7, E11.8, E13.21, E13.22, E13.29, E13.31, E13.311, E13.319, E13.32, E13.321, E13.3211, E13.3212, E13.3213, E13.3219, E13.329, E13.3291, E13.3292, E13.3293, E13.3299, E13.33, E13.331, E13.3311, E13.3312, E13.3313, E13.3319, E13.339, E13.3391, E13.3392, E13.3393, E13.3399, E13.34, E13.341, E13.3411, E13.3412, E13.3413, E13.3419, E13.349, E13.3491, E13.3492, E13.3493, E13.3499, E13.35, E13.351, E13.3511, E13.3512, E13.3513, E13.3519, E13.352, E13.3521, E13.3522, E13.3523, E13.3529, E13.353, E13.3531, E13.3532, E13.3533, E13.3539, E13.354, E13.3541, E13.3542, E13.3543, E13.3549, E13.355, E13.3551, E13.3552, E13.3553, E13.3559, E13.359, E13.3591, E13.3592, E13.3593, E13.3599, E13.36, E13.37, E13.37X1, E13.37X2, E13.37X3, E13.37X9, E13.39, E13.40, E13.41, E13.42, E13.43, E13.44, E13.49, E13.610, E13.621, G63.2, H28.0, H34.81, H34.811, H34.8110, H34.8111, H34.8112, H34.812, H34.8120, H34.8121, H34.8122, H34.813, H34.8130, H34.8131, H34.8132, H34.819, H34.8190, H34.8191, H34.8192, H34.83, H34.831, H34.8310, H34.8311, H34.8312, H34.832, H34.8320, H34.8321, H34.8322, H34.833, H34.8330, H34.8331, H34.8332, H34.839,	**ICD-10:** E11.0-E11.8, E16.0, H28.0, H36.0, G59.0, G63.2, G73.0, G99.0, I79.2, M14.2, M14.6, N08.3	**ICD-9:** 583.81, 250.4x, 362.0x, 250.5x, 366.41, 365.44, 362.83, 707.1x, 585.3, 585.4, 585.5, 585.6, 582.xx, 583.xx, 585.1, 585.2, 585.9, 586.xx, 587.xx, 642.1x, 250.4x, 250.40, 250.41, 250.42, 250.43, 403.xx, 404.xx, 572.4x, 580.xx, 584.xx, 580.0x, 580.4x, 580.89, 580.9x, 582.4x, 642.1x, 791.2x, 791.3x

© 2023 Cesta CE, et al. *JAMA Intern Med.*

Condition	US (MarketScan Research Database)	Nordic Registers	Israel (Maccabi Health Services Database)
	H34.8390, H34.8391, H34.8392, H35.31, H35.311, H35.3110, H35.3111, H35.3112, H35.3113, H35.3114, H35.312, H35.3120, H35.3121, H35.3122, H35.3123, H35.3124, H35.313, H35.3130, H35.3131, H35.3132, H35.3133, H35.3134, H35.319, H35.3190, H35.3191, H35.3192, H35.3193, H35.3194, H35.32, H35.321, H35.3210, H35.3211, H35.3212, H35.3213, H35.322, H35.3220, H35.3221, H35.3222, H35.3223, H35.323, H35.3230, H35.3231, H35.3232, H35.3233, H35.329, H35.3290, H35.3291, H35.3292, H35.3293, H36.0, H42., H43.1, H43.10, H43.11, H43.12, H43.13, I79.2, L97.201, L97.202, L97.203, L97.204, L97.205, L97.206, L97.208, L97.209, L97.211, L97.212, L97.213, L97.214, L97.215, L97.216, L97.218, L97.219, L97.221, L97.222, L97.223, L97.224, L97.225, L97.226, L97.228, L97.229, L97.301, L97.302, L97.303, L97.304, L97.305, L97.306, L97.308, L97.309, L97.311, L97.312, L97.313, L97.314, L97.315, L97.316, L97.318, L97.319, L97.321, L97.322, L97.323, L97.324, L97.325, L97.326, L97.328, L97.329, L97.401, L97.402, L97.403, L97.404, L97.405, L97.406, L97.408, L97.409, L97.411, L97.412, L97.413, L97.414, L97.415, L97.416, L97.418, L97.419, L97.421, L97.422, L97.423, L97.424, L97.425, L97.426, L97.428, L97.429, L97.501, L97.502, L97.503, L97.504, L97.505, L97.506, L97.508, L97.509, L97.511, L97.512, L97.513, L97.514, L97.515, L97.516, L97.518, L97.519, L97.521, L97.522, L97.523, L97.524, L97.525, L97.526, L97.528, L97.529, L97.801, L97.802, L97.803, L97.804, L97.805, L97.806, L97.808, L97.809, L97.811, L97.812, L97.813, L97.814, L97.815, L97.816, L97.818, L97.819, L97.821, L97.822, L97.823, L97.824, L97.825, L97.826, L97.828, L97.829, L97.901, L97.902, L97.903, L97.904, L97.905, L97.906, L97.908, L97.909, L97.911, L97.912, L97.913, L97.914, L97.915, L97.916, L97.918, L97.919, L97.921, L97.922, L97.923, L97.924, L97.925, L97.926, L97.928, L97.929, M14.2, M14.671, M14.672, M14.679, N08.3, Z86.31		
Cardiovascular Disease	**ICD-9:** 428.0, 428.1, 428.20, 428.21, 428.22, 428.23, 428.30, 428.31, 428.32, 428.33, 428.40, 428.41, 428.42, 428.43, 428.9, 410.00, 410.01, 410.02, 410.10, 410.11, 410.12, 410.20, 410.21, 410.22, 410.30, 410.31, 410.32, 410.40, 410.41, 410.42, 410.50, 410.51, 410.52, 410.60, 410.61, 410.62, 410.70, 410.71, 410.72, 410.80, 410.81, 410.82, 410.90, 410.91, 410.92, 411.0, 411.1, 411.81, 411.89, 412, 413.0, 413.1, 413.9, 414.00, 414.01, 414.02, 414.03, 414.04, 414.05, 414.06, 414.07, 414.10, 414.11, 414.12, 414.19, 414.2, 414.3, 414.4, 414.8, 414.9, 427.31, 427.32<br>**ICD-10:** I20.0, I20.1, I20.8, I20.9, I21.01, I21.02, I21.09, I21.11, I21.19, I21.21, I21.29, I21.3, I21.4, I21.9, I21.A1, I21.A9, I22.0, I2.21, I2.22, I2.28, I2.29, I24.0, I24.1, I2.48, I2.49, I25.10, I25.110, I25.111, I25.118, I25.119, I25.2, I25.3, I2.541, I25.42, I2.55, I25.6, I25.700, I25.701, I25708, I25.709, I25.710, I25.711, I25.718, I25.719, I25.720, I25.721, I25.728, I25.729, I25.730, I25731, I25.738, I257.39, I25.750, I25.751, I25.758, I25.759, I25.760, I25.761, I25.768, I25.769, I25790, I25.791, I25.798, I25.799, I25.810, I25.811, I25.812, I25.82, I25.83, I25.84, I25.89, I25.9, I480, I481, I48.2, I48.3, I48.4, I48.91, I48.92, I50.1, I50.20, I50.21, I50.22, I50.23, I50.30, I50.31, I50.32, I50.33, I50.40, I5.041, I50.42, I50.43, I50.810, I50.811, I50.812, I50.813, I50.814, I50.82, I50.83, I50.84, I508.9, I50.9	**ICD-10:** I20-I25, I48, I50, I61-I66, I69.1-I69.4, I70-I72, I73.9, I74	At least two diagnoses (ICD-9) for one of the following cardiovascular disease groups: ischemic heart disease (410.x–414.x), congestive heart failure (428.x), vascular disease and atherosclerosis (440.x-447.x, 557.x), arrhythmias and conduction disorders (426.x-427.x), pericardium and endocardium diseases (423.x-424.x); Chronic rheumatic heart disease(391.x, 393.x-398.x), cardiomyopathic diseases (425.x), hypertensive heart disease (402.x,404.x), inflammatory heat conditions (393.x, 398.x,420.x-422.x) pulmonary heart disease (415.x-416.x); other heart conditions (429.x, 794.x, 785.x); Cerebrovascular disease (430.x–438.x); congenital heart anomalies (745.x-747.x); Benign and malignant heart neoplasm (212.7, 164.1)
Polycystic ovary syndrome	**ICD-9:** 256.4x<br>**ICD-10:** E28.2x	**ICD-10:** E28.2	**ICD-9:** 256.4x
**Prescription medications**	≥1 dispensation in maternal record from LMP-90 to LMP+90	≥1 dispensation in the prescribed drug registers from LMP-90 to LMP+97	Assessment period = LMP-180 to LMP+90
Antihypertensives	NDC codes	ATC codes:C02 (antihypertensives), C03 (diuretics), C07 (beta blockers), C08 (calcium channel blockers)	ATC codes:C02 (antihypertensives), C03 (diuretics), C07 (beta blockers), C08 (calcium channel blockers)
Lipid modifying agents	NDC codes	ATC codes: C10	ATC codes: C10

Abbreviations: ATC code, Anatomical Therapeutic Chemical code; BMI, body mass index; Cat, category; ICD, International Classification of Diseases; LMP, last menstrual period; n/a, not available; NDC, national drug code.

© 2023 Cesta CE, et al. ***JAMA Intern Med.***

Supplementary Table 6. Mean and Median HbA_{1c} for the full study population and women with pre-gestational type 2 diabetes mellitus with different periconceptional antidiabetic medication use in a subset of the US MarketScan and Israel Maccabi Health Services databases

	US - MarketScan				Israel -MHS			
	n [1]	% of total population with HbA1c values	mean (SD)	median (IQR)	n [1]	% of total population with HbA1c values	mean (SD)	median (IQR)
All pregnancies	6286	0.9%	5.4 (0.7)	5.3 (0.4)	31686	8.5%	5.2 (0.4)	5.2 (0.4)
T2DM	397	4.3%	6.7 (1.6)	6.1 (1.5)	575	58.2%	6.0 (0.9)	5.8 (0.8)
No ADM	127	2.6%	5.9 (1.1)	5.7 (0.8)	165	53.1%	5.8 (0.7)	5.7 (0.7)
Metformin only	87	6.1%	6.3 (1.1)	6.0 (1.1)	90	61.6%	5.9 (0.8)	5.8 (0.7)
Insulin	64	5.1%	7.8 (2.0)	7.2 (2.9)	253	60.0%	6.0 (0.8)	5.8 (0.8)
Sulfonylureas	81	8.1%	7.1 (1.7)	6.5 (1.8)	60	52.2%	6.0 (1.1)	5.7 (0.6)
DDP-4 inhibitors	23	7.2%	7.8 (1.8)	7.6 (2.6)	55	68.8%	6.7 (1.1)	6.5 (1.6)
GLP1 receptor agnists	35	6.2%	7.0 (1.7)	6.7 (2.6)	19	44.2%	6.0 (0.9)	5.8 (0.9)
SGLT2 inhibitors	13	7.6%	8.0 (2.5)	7.4 (3.2)	25	71.4%	6.7 (1.2)	6.6 (1.5)

Abbreviations: ADM, antidiabetic medication; T2DM, type 2 diabetes mellitus; DPP4, Dipeptidyl peptidase 4; GLP-1, Glucagon-like peptide-1; SGLT2, Sodium-glucose co-transporter 2; SD, standard deviation; IQR, interquartile range.
[1] number of women with a recorded HbA1c value

For the US:
Only a subsample (around 7%) of MarketScan enrollees has laboratory data available in the Research Database, i.e., a higher proportion of pregnancies had HbA1c tests, but they are not available to investigators.
Pregnancies with at least one LOINC code for HbA_{1c} test within LMP-90 to LMP+90 were identified.
Logical Observation Identifier Names and Codes (LOINC) for HbA_{1c} test results: 4548-4, 17856-6, 17855-8, 4549-2, 55454-3.
Any lab values outside the range allowed (2-20 units), re-coded as missing.

For Israel:
Pregnancies with at least one HbA_{1c} test within LMP-90 to LMP+90 were identified.

For both US and Israel:

- If more than 1 HbA_{1c} value during the assessment period, the one closest to the LMP was selected
- If more than 1 HbA_{1c} test was taken on the same date, the average of values was calculated
- If any 2 HbA_{1c} tests are of equal distance in time (days) from LMP, the average of the values was calculated

© 2023 Cesta CE, et al. ***JAMA Intern Med.***

Supplementary Table 7. Prevalence and 95% confidence intervals of any and cardiac major congenital malformation in live-born infants in the full pregnancy cohort and born to women with type 2 diabetes mellitus with periconceptional use of no antidiabetic medication, metformin only, insulin, or 2nd-line non-insulin antidiabetic medications

		Any Major Congenital Malformation		Cardiac Malformations	
	Number of exposed	Number of cases	Pooled prevalence, % (95% CI)	Number of cases	Pooled prevalence, % (95% CI)
**All Pregnancies**	3514865	132283	3.76 (3.74-3.78)	45988	1.31 (1.30-1.32)
**All T2DM**	51826	2584	5.28 (5.04-5.53)	1115	2.25 (2.09-2.41)
**No ADM**	36678	1602	4.77 (4.42-5.12)	858	2.30 (2.07-2.53)
**Metformin only**	7440	377	5.32 (4.68-5.95)	141	2.04 (1.62-2.46)
**Insulin**	5078	400	7.83 (7.09-8.57)	212	4.20 (3.64-4.76)
**Sulfonylureas**	1362	121	9.71 (6.80-12.62)	50	4.85 (2.65-7.04)
**DPP4 inhibitors**	687	50	6.14 (4.15-8.14)	24	3.26 (1.75-4.78)
**GLP-1 receptor**	938	75	8.23 (5.58-10.88)	23	3.22 (1.43-5.01)
**agnists SGLT2 inhibitors**	335	30	7.04 (3.35-10.73)	15	3.88 (1.01-6.75)

Abbreviations: ADM, antidiabetic medication; T2DM, type 2 diabetes mellitus; DPP4, Dipeptidyl peptidase 4; GLP-1, Glucagon-like peptide-1; SGLT2, Sodium-glucose co-transporter 2.

© 2023 Cesta CE, et al. ***JAMA Intern Med.***

Supplementary Table 8. Crude and adjusted relative risks and 95% confidence intervals for any and cardiac major congenital malformations in infants born to women with type 2 diabetes mellitus and periconceptional use of 2nd-line non-insulin antidiabetic medication compared with insulin, presented separately for the Nordic countries (pooled Finland, Iceland, Norway, Sweden), US MarketScan, and Israel Maccabi Health Services databases

	Any Major Congenital Malformation			Cardiac Malformation		
	No. exposed cases / No. exposed (%)	Crude Relative Risk (95%CI)	Adjusted Relative Risk (95% CI) [a]	No. exposed cases / No. exposed (%)	Crude Relative Risk (95%CI)	Adjusted Relative Risk (95% CI) [a]
**Nordic**						
Insulin	256/3269 (7.8)	reference	reference	151/3269 (4.6)	reference	reference
Sulfonylureas	20/198 (10.1)	1.29 (0.84-1.99)	1.32 (0.85-2.05)	11/198 (5.6)	1.20 (0.66-2.18)	1.19 (0.65-2.16)
DPP4 inhibitors	14/266 (5.3)	0.67 (0.40-1.13)	0.67 (0.39-1.14)	8/266 (3.0)	0.65 (0.32-1.31)	0.64 (0.32-1.30)
GLP-1 receptor agnists	16/214 (7.5)	0.95 (0.57-1.59)	1.02 (0.60-1.73)	8/214 (3.8)	0.81 (0.38-1.74)	0.91 (0.41-2.04)
SGLT2 inhibitors	5/82 (6.1)	0.78 (0.33-1.82)	0.85 (0.35-2.02)	<5/82	0.79 (0.26-2.40)	0.93 (0.31-2.79)
**US-MarketScan**						
Insulin	115/1387 (8.3)	reference	reference	48/1387 (3.5)	reference	reference
Sulfonylureas	90/1049 (8.6)	1.04 (0.78-1.38)	1.10 (0.82-1.47)	35/1049 (3.3)	0.96 (0.62-1.49)	0.97 (0.62-1.51)
DPP4 inhibitors	32/341 (9.4)	1.15 (0.76-1.72)	1.18 (0.79-1.78)	13/341 (3.8)	1.11 (0.59-2.06)	1.12 (0.60-2.08)
GLP-1 receptor agnists	53/681 (7.8)	0.93 (0.67-1.31)	0.85 (0.59-1.22)	14/681 (2.1)	0.59 (0.32-1.07)	0.57 (0.29-1.09)
SGLT2 inhibitors	23/218 (10.6)	1.30 (0.81-2.09)	1.05 (0.65-1.70) [b]	11/218 (5.0)	1.48 (0.76-2.90)	1.21 (0.61-2.40) [b]
**Israel - MHS**						
Insulin	29/422 (6.9)	reference	reference	13/422 (3.1)	reference	reference
Sulfonylureas	11/115 (9.6)	1.39 (0.72-2.70)	1.33 (0.68-2.60)	4/115 (3.5)	1.13 (0.37-3.40)	1.14 (0.39-3.34)
DPP4 inhibitors	4/80 (5.0)	0.73 (0.26-2.02)	0.66 (0.24-1.84)	3/80 (3.8)	1.22 (0.35-4.18)	1.10 (0.32-3.78)
GLP-1 receptor agnists	6/43 (14.0)	2.03 (0.89-4.41)	1.69 (0.64-4.44)	1/43 (2.3)	0.75(0.10-5.63)	0.64 (0.09-4.59)
SGLT2 inhibitors	2/35 (5.7)	0.83 (0.21-3.35)	0.73 (0.18-2.98)	1/35 (2.9)	0.93 (0.12-6.90)	0.83 (0.11-6.50)

Counts <5 are not shown for data privacy policies in the Nordics

[a] Adjusted for birth year, maternal age, obesity, and specific Nordic country (in the pooled Nordic cohort only)

[b] Adjusted for birth year and obesity

Abbreviations: CI, confidence intervals; DPP4, Dipeptidyl peptidase 4; GLP-1, Glucagon-like peptide-1; SGLT2, Sodium-glucose co-transporter 2.

© 2023 Cesta CE, et al. *JAMA Intern Med.*

Supplementary Table 9. Crude relative risks and 95% confidence intervals for any and cardiac major congenital malformation in infants born to women with type 2 diabetes mellitus and first trimester use of 2nd-line non-insulin antidiabetic medications compared with insulin, presented meta-analyzed and separately for the Nordic countries (pooled Finland, Iceland, Norway, Sweden), US MarketScan, and Israel Maccabi Health Services databases

	Any Major Congenital Malformation		Cardiac Malformation	
	No. exposed cases / No. exposed (%)	Crude Relative Risk (95%CI)	No. exposed cases / No. exposed (%)	Crude Relative Risk (95%CI)
**All**				
Insulin	412/5194 (7.8 [a])	reference	218/5194 (4.2 [a])	reference
Sulfonylureas	88/1070 (9.1 [a])	1.02 (0.79-1.32)	35/1070 (3.2 [a])	0.89 (0.6-1.33)
DPP-4 inhibitors	27/400 (5.8 [a])	0.82 (0.55-1.23)	10/400 (1.9 [a])	0.68 (0.35-1.31)
GLP-1 receptor agnists	38/461 (6.7 [a])	1.03 (0.73-1.47)	8/461 (1.6 [a])	0.46 (0.22-0.94) [b]
SGLT2 inhibitors	15/181 (4.3 [a])	1.20 (0.69-2.11) [b]	7/181 (1.2 [a])	1.56 (0.70-3.50) [c]
**Nordic**				
Insulin	245/3164 (7.7)	reference	147/3164 (4.7)	reference
Sulfonylureas	9/98 (7.7)	1.19 (0.63-2.24)	<5/98	0.66 (0.21-2.03)
DPP-4 inhibitors	7/134 (5.2)	0.67 (0.32-1.40)	<5/134	0.32 (0.08-1.28)
GLP1 receptor agnists	5/110 (4.6)	0.59 (0.25-1.40)	<5/110	0.39 (0.1-1.56)
SGLT2 inhibitors	<5/36	0.36 (0.05-2.49)	0/36	no exposed cases
**US-MarketScan**				
Insulin	136/1583 (8.6)	reference	58/1583 (3.7)	reference
Sulfonylureas	68/870 (7.8)	0.90 (0.67-1.22)	28/870 (3.2)	0.87 (0.55-1.39)
DPP4 inhibitors	18/224 (8.0)	0.93 (0.56-1.55)	7/224 (3.1)	0.85 (0.38-1.88)
GLP-1 receptor agnists	30/331 (9.1)	1.06 (0.70-1.61)	6/331 (1.8)	0.49 (0.21-1.14)
SGLT2 inhibitors	14/125 (11.2)	1.34 (0.75-2.41)	7/125 (5.6)	1.56 (0.70-3.50)
**Israel-MHS**				
Insulin	31/447 (6.9)	reference	13/447 (2.9)	reference
Sulfonylureas	11/102 (6.9)	1.56 (0.81-2.99)	4/102 (3.9)	1.35 (0.45-4.05)
DPP4 inhibitors	2/42 (4.8)	0.69 (0.17-2.77)	1/42 (2.4)	0.82 (0.11-6.11)
GLP-1 receptor agnists	3/20 (15.0)	2.16 (0.72-6.45)	0/20	no exposed cases
SGLT2 inhibitors	0/20	no exposed cases	0/20	no exposed cases

Counts <5 are not shown for data privacy policies in the Nordics

[a] Standardized prevalence

[b] No exposed cases in Israel, includes only Nordic and US estimates

[c] No exposed cases in Israel or the Nordics

Abbreviations: CI, confidence intervals; DPP4, Dipeptidyl peptidase 4; GLP-1, Glucagon-like peptide-1; SGLT2, Sodium-glucose co-transporter 2.

© 2023 Cesta CE, et al. *JAMA Intern Med.*

Supplementary Methods 1. Description of Nordic Data and Ethical Approval Information.

Description of the Nordic databases

Similar health and social registers exist in Finland, Iceland, Norway, and Sweden, which are linkable by a personal identity number assigned to all residents of each country. The Nordic countries all have publicly funded healthcare systems with reporting to the registers mandated by law.

For more information on the Nordic Health registers, see the publication by Laugesen and colleagues.(4)

Finland

- The **Finnish Medical Birth Register** was established in 1987. The registry includes data on all live births and stillbirths of at least 500 grams or 22 completed gestational weeks, as well as data on mothers.
- **Register of Reimbursed Drug Purchases and Register of Medical Special Reimbursements**. Nationwide databases on reimbursed drug purchases available from 1995. If an individual is entitled to special reimbursement, then drug refill is accompanied with indication for which the prescription was reimbursed.
- The **Finnish Patient Register - Care Register for Health care (HILMO)** contains dates of hospitalizations and visits, all diagnoses (ICD codes) and surgical procedures (NCSP codes) from inpatient care in all hospitals since 1967 and outpatient specialist care in public hospitals since 1998.
- **Finnish Register of Congenital Anomalies** contains all malformations (including terminations and stillbirths) identified within one year of birth, which have been validated based on hospital records. (https://thl.fi/en/web/thlfi-en/statistics/information-on-statistics/quality-descriptions/congenital-anomalies)

Iceland

- **Icelandic Medical Birth Register**: Since 1981, mandatory registration of all live- and stillbirths of at least 500 grams or 22 completed gestational weeks.
- **Icelandic Prescription Medicines Register**: Since 2003, mandatory registration of all outpatient drugs dispensed in Iceland. Prescription drugs administered to individuals in nursing homes are included after from 2011 onwards.
- The **Icelandic Patient Register:** Contains dates of hospitalizations and visits, all diagnoses (ICD codes) and surgical procedures (NCSP codes) from hospital inpatient stays since 1999 and outpatient wards since 2010.
- **Statistics Iceland**: Highest obtained level of education categorized on an annual basis according to the International Standard Classification of Education (ISCED).

Norway

- **Medical Birth Registry of Norway (MBRN):** Since 1967, mandatory registration of all live- and stillbirths from 16 completed gestational weeks. Includes all pregnancy terminations after 12 weeks for fetal anomaly (TOPFA) since 1999. Includes data from Neonatal Intensive Care Units, including neonatal and major congenital malformation diagnoses, for all infants transferred to such units after birth since 1999.
- **Norwegian Prescription Database (NorPD):** Since January 2004, mandatory registration of all drugs dispensed in Norway. Dispensed drugs are recorded according to the Anatomical Therapeutic Chemical (ATC) classification system, with the date of dispensation, the strength, and quantity Since 2008, includes ICD-10 or ICPC-2 diagnosis for drugs dispensed for chronic conditions. Before 2008, another reimbursement coding system was used. Drugs administered in hospitals or nursing homes are not included.
- **Norwegian Patient Registry (NPR):** Since 2008, dates, ICD-10 diagnoses, and procedures (NCSP codes) from all public specialists' health-care services (both inpatient and outpatient care), including private institutions contracted to the regional health authorities.
- **Norwegian Population Register (National Registry):** Migration/moving, marriage and cohabitation

Sweden

- **The Swedish Medical Birth Register** covers since 1973, 99% of all live- and stillbirths from gestational week 22. Before July 1,2008 stillbirths occurring from week 28 only were included. Data is collected from antenatal care clinics, delivery units and pediatric examination of the infant.
- **The Swedish Prescribed Drug Register** was established in July 2005 and contains complete national data on all pharmaceuticals dispensed on prescription. Drugs are recorded according to the Anatomical Therapeutic Chemical (ATC)

classification system, with the date of dispensation, the strength, and quantity. Drugs administered in hospitals or nursing homes are not included.

- **The Swedish Patient Register** contains dates of hospitalizations and visits, all diagnoses (ICD codes) and surgical procedures (NCSP codes) from inpatient stays. The reporting started in 1964 and since 1987, the register has covered all public, inpatient care in Sweden. Since 2001 both public and private care is included in the register and since 2002 there is almost full coverage of all outpatient hospital visits.

Ethical boards providing approval for data use & approval numbers

Country	Ethical Board	Approval Number
Finland	No Ethical Board approval needed.	THL/1551/6.02.00/2018, THL/1673/5.05.00/2019, Kela 117/522/2019
Iceland	National Bioethics Committee	VSNb2018060017/03.01
Norway	Regional Committee for Medical Research Ethics South/East Norway<br>The Norwegian Data Inspectorate in Norway	2017/2546/REC South-East<br>17/02068/Norwegian Data Inspectorate
Sweden	Swedish Ethical Review Authority (Etikprövningsmyndigheten)	dnr 2015/1826-31/2, 2017/2238-32, 2018/1790-32, 2018/2211-32, 2022-04004-02

© 2023 Cesta CE, et al. ***JAMA Intern Med.***

Supplementary Methods 2: Pre-gestational Diabetes Algorithms

a. **US cohort –** Pregnant women with a pre-gestational T2DM were identified using a validated algorithm developed by Wood et al.,(2021)(5)

Summary of algorithm:

1) *Identify any diabetes*
2) *Evaluate for pre-gestational diabetes*
3) *Rule out Type 1 diabetes*
4) *Type 2 diabetes: Meets ANY 1 criterion LMP-180 to LMP+90:*
   - *≥2 filled prescriptions of non-metformin type 2 antidiabetic drug (i.e., excludes insulin and the few oral drugs occasionally used for type 1 DM)*
   - *0 filled prescriptions for insulin*
   - *(≥ 1 filled prescription for insulin) + (≥2 type 2 diabetes codes) + (≤1 type 1 diabetes codes)*

ICD-9-CM	ICD-10	Definition
250.x1, 250.x3	O24.0x, O24.0xx, E10.x, E10.xx, E10.xxx, E10.xxxx	**Type 1 pre-gestational diabetes**
250.x0, 250.x2	O24.1x, O24.1xx, E11.x, E11.xx, E11.xxx, E11.xxxx	**Type 2 pre-gestational diabetes**

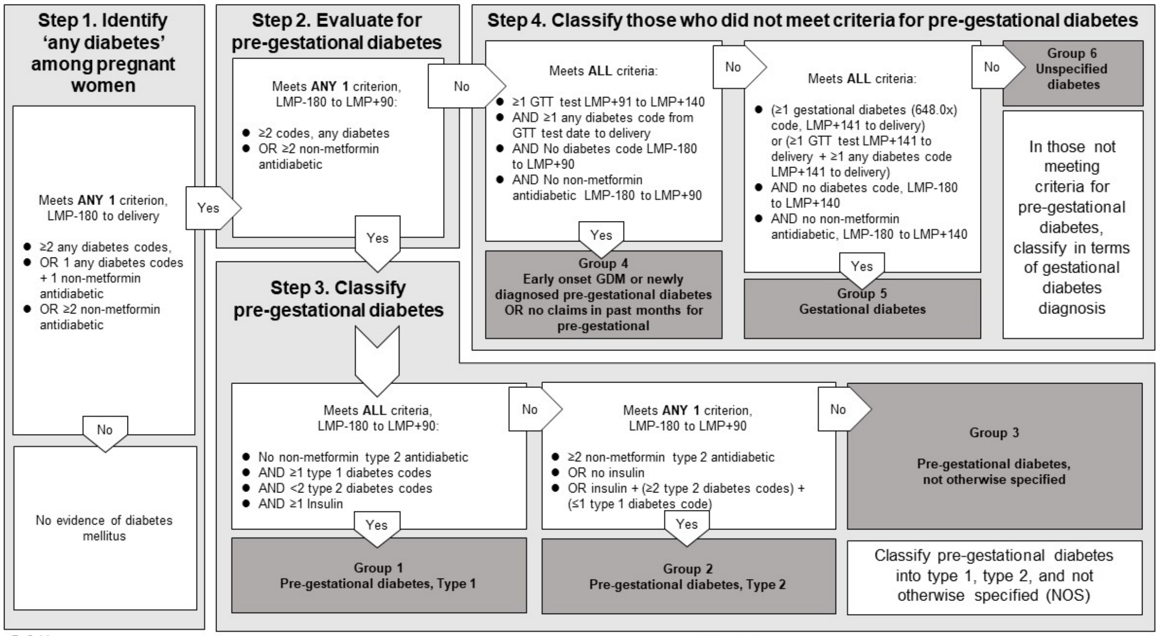

Definitions
Non metformin antidiabetic =insulin or oral non-metformin antidiabetic; **Non metformin type 2 antidiabetics** = those used only for type 2 (i.e. excludes insulin and the few orals occasionally used for type 1

Abbreviations
GTT=glucose tolerance test; LMP=last menstrual period (estimated); NOS=not otherwise specified

© 2023 Cesta CE, et al. *JAMA Intern Med.*

b. **Nordic** – Algorithm used for pooled data from Finland, Iceland, Norway, and Sweden

Summary of algorithm:

1) *Identify any diabetes (LMP-1 year to delivery)*
2) *Evaluate for pre-gestational diabetes (LMP-1 year to LMP+90) including medication*
3) *Rule out Type 1 diabetes and other types of pre-gestational diabetes*
4) *Type 2 diabetes are the remaining pre-gestational diabetes cases*

Step 1. Identify any diabetes mellitus among pregnant women

Meets any criterion, LMP-1 year to delivery*

- **MBR (chronic_diabetes, gest_diabetes) – based on check boxes in Norway, Special Refund Entitlement Register in Finland, and ICD-10 codes in all**
- ≥1 diabetescode in **NPR** (ICD-10: E10-E14, O24)
- ≥1 diabetescode in **primary care**** (ICD-10: E10-E14, O24, or ICPC-2: T89-T90, W85)
- ≥1 antidiabetic drug prescription in **PDR***** (ATC A10)

* If the year before LMP includes another pregnancy, ignore diagnoses or prescriptions from a prior pregnancy (applies to steps 1-4)

** Primary care only available for Norway (from 2006) and Finland (from 2012/2013)

*** Exclude if they have zero diabetes diagnoses and metformin as the only antidiabetic, and ART pregnancy (see below) or polycystic ovary syndrome (E282) recorded in NPR/MBR

Step 2. Evaluate for pregestational diabetes mellitus

Meets any criterion:

- **MBR (chronic_diabetes = 1)**
- ≥1 code for any diabetes in **NPR** or **primary care** LMP-1 year to LMP+90 days
- ≥1 antidiabetic drug (A10) prescription in **PDR**, LMP-1 year to LMP+90 days
- ≥1 code for pregestational diabetes mellitus in **NPR** or **primary care** LMP+91 days to delivery

Step 3. Classify pregestational diabetes mellitus

3A. Pregestational diabetes mellitus, type 1 (pG1)

Meets ALL criteria (third criterion ignored if DIABETES_MELLITUS=1 in the second)

- ≥2 insulin prescriptions, LMP-1 year to LMP+90 days
- ≥1 type 1 code in **NPR** (ICD-10: E10, O24.1) LMP-1 year to delivery, or DIABETES_MELLITUS = 1 in **MBRN**
- If codes for both type 1 and 2 and/or other in **NPR** => majority type 1 codes

3B. Pregestational diabetes mellitus, other types (pg0)

Meets ALL criteria

- ≥1 code other than type 1 or 2 in **NPR** (ICD-10: E12-E13, O24.2, O24.3), LMP-1 year to delivery
- If codes for type 1 or 2 are also present => majority codes for other types

3C. Pregestational diabetes mellitus, type 2 (pG2)

Remaining pregestational diabetes mellitus

ART

Meets ANY criterion

- Checkbox in MBRN/MBRS
- ICD-10 code Z311, Z312, Z313 or N98 between LMP-120 and LMP+30 days
- GnRH-analogue + gonadotropin + HCG between LMP-120 and LMP+30 days
  - GnRH: ATC codes H01CA02, H01CC01, H01CC02, L02AE01 or L02AE03
  - Gonadotropin: ATC codes G03GA02, G03GA04, G03GA05, G03GA06, G03GA09 or G03GA30
  - HCG: ATC codes G03GA01 or G03GA08

Step 4. Classify those who did not meet criteria for pregestational diabetes mellitus

4A. Gestational diabetes mellitus (G)

Meets ALL criteria

- ≥1 gestational diabetes code in **NPR/primary care** LMP+91 days to delivery or **MBR** (gest_diabetes = 1)
- No antidiabetic drug prescription before LMP-1 year to LMP+90 days

4B. Diabetes mellitus NOS (N)

All remaining diabetes mellitus

Step 5.

Check for pregestational diabetes mellitus in a prior pregnancy:

- For N or G or pG0 pregnancy, if they had pG2 in any prior pregnancy, reclassify as pG2
- For N, G, pG, if they had a pG1 in any prior or future pregnancy, reclassify as pG1
- For no diabetes mellitus, if they had pG2 in a prior pregnancy, reclassify as pG2

ICD-10 and ICPC-2 codes

Pregestational diabetes mellitus, type 1: ICD-10: O240, E10

Pregestational diabetes mellitus, type 2: ICD-10: O241, E11

Pregestational diabetes mellitus, other: ICD-10: O242, O243, E12, E13

Gestational diabetes mellitus: ICD-10: O244, O249; ICPC-2: W85

Abbreviations

ATC: Anatomical Therapeutic Chemical classification system

GnRH: Gonadotropin-Releasing Hormone

HCG: Human chorionic gonadotropin

ICD-10: International Classification of Diseases, 10th Revision

ICPC-2: International Classification of Primary Care, 2nd Edition

LMP: First day of last menstrual period before pregnancy

MBR: Medical Birth Register

MBRN: Medical Birth Register of Norway

MBRS: Medical Birth Register of Sweden

NPR: National Patient Register (specialist health care, in- and outpatient)

PDR: Prescribed Drug Register

© 2023 Cesta CE, et al. *JAMA Intern Med.*

c. **Israel** – pregnant women with pre-gestational T2DM were identified by inclusion into the Maccabi Health Services diabetes register,(6) according to the following criteria:

Criteria for the diabetes registry

1. HbA1c ≥ 7.25%
2. At least two random glucose tests ≥200 mg/dl with a minimum gap of 30 days between tests.
3. Oral drugs: Purchase of at least two oral medications for diabetes in a three-month period, with one of the following: A) at least one glucose test above 125 mg/dl; B) HbA1c ≥ 6.5%.
   - . Purchase of oral medications on the same day counts as only one purchase
   - . A woman of childbearing age (18-55 years) with a GCT or GTT test (no matter the value), whose last purchase of an oral antidiabetic drug was within less than four months from the data of the test does not enter the registry (added to the gestational diabetes registry).
4. Insulin: Purchase of at least two insulin medications in a three-month period.
   - a. A patient who entered the registry based on dispensing of insulin alone and who did not have additional dispensing records for insulin in the 6 consecutive months following the initial dispensing date is removed from the registry.
   - b. Purchase of multiple insulin drugs on the same day counts as only one purchase
   - c. A woman of childbearing age (18-55 years) with a GCT or GTT test (no matter the value), whose last purchase date of insulin was within less than four months from the date of the test does not enter the registry (added to the gestational diabetes registry).
5. Diabetes related diagnoses: diagnosis recorded by a family physician/ pediatrician, diabetes consultant, endocrinologist, or ophthalmologist, with one of the following:
   - a. A. HbA1c≥ 6.5% within six months before or after diagnosis
   - b. Two glucose tests > 125 mg/dl, one six months before or after diagnosis and one at any point in time.

Determining type of diabetes:

- o Type 1 diabetes –
  - . Added to the registry at age ≤ 26
  - . Purchased insulin 5 or more times, and did not purchase other oral medications, or if other oral medications were purchased, first purchase occurred after at least 5 consecutive insulin purchases and after at least 1 year in the registry.
- o Type 2 diabetes –
  - . Patients in the registry who did not purchase insulin for at least 3 years from the time they entered the registry.
  - . Patients who discontinued insulin and started therapy with other oral medications for diabetes who were treated with oral medications for at least 3 years after discontinuation of insulin (last oral purchase date - last insulin purchase date> 3 years).
  - . Patients who received both insulin and oral medications for diabetes, conditional that the oral medications were initially received at least 3 years before insulin onset (first insulin purchase date - first oral purchase date> 3 years).

© 2023 Cesta CE, et al. *JAMA Intern Med.*

References

1. Cohen JM, Alvestad S, Cesta CE, Bjørk MH, Leinonen MK, Nørgaard M, et al. Comparative Safety of Antiseizure Medication Monotherapy for Major Malformations. Ann Neurol. 2023;93(3):551-62.
2. Palmsten K, Huybrechts KF, Kowal MK, Mogun H, Hernández-Díaz S**.** Validity of maternal and infant outcomes within nationwide Medicaid data. Pharmacoepidemiol Drug Saf. 2014;23(6):646-55.
3. He M, Huybrechts KF, Dejene SZ, Straub L, Bartels D, Burns S, et al. Validation of algorithms to identify adverse perinatal outcomes in the Medicaid Analytic Extract database. Pharmacoepidemiol Drug Saf. 2020;29(4):419-26.
4. Laugesen K, Ludvigsson JF, Schmidt M, Gissler M, Valdimarsdottir UA, Lunde A, et al. Nordic Health Registry-Based Research: A Review of Health Care Systems and Key Registries. Clin Epidemiol. 2021;13:533-54.
5. Wood ME, Chen ST, Huybrechts KF, Bateman BT, Gray KJ, Seely EW, et al. Validation of a claims-based algorithm to identify pregestational diabetes among pregnant women in the United States. Epidemiology. 2021.
6. Chodick G, Heymann AD, Shalev V, Kookia E**.** The epidemiology of diabetes in a large Israeli HMO. Eur J Epidemiol. 2003;18(12):1143-6.

© 2023 Cesta CE, et al. *JAMA Intern Med.*
