# Supplementary material for: Safety of GLP-1 Receptor Agonists and Other Second-Line Antidiabetics in Early Pregnancy
Source: JAMA Intern Med. 2023 Dec 11;184(2):144–52. doi: 10.1001/jamainternmed.2023.6663 (PMC10714281; doi:10.1001/jamainternmed.2023.6663)
Supplement: Supplement 2. — Data Sharing Statement [file jamainternmed-e236663-s002.pdf]

Data Sharing Statement

Cesta. Safety of GLP-1 Receptor Agonists and Other Second-Line Antidiabetics in Early Pregnancy. *JAMA Intern Med*. Published December 11, 2023.
doi:10.1001/jamainternmed.2023.6663

Data
Data available: No

Additional Information
Explanation for why data not available: Due to data privacy laws, the data cannot be made publicly available.
